# SpCas9-HF1 enhances accuracy of cell cycle-dependent genome editing by increasing HDR efficiency, and by reducing off-target effects and indel rates

**DOI:** 10.1016/j.omtn.2024.102124

**Published:** 2024-01-23

**Authors:** Daisuke Matsumoto, Erina Matsugi, Kanae Kishi, Yuto Inoue, Kiyomi Nigorikawa, Wataru Nomura

**Affiliations:** 1 Graduate School of Biomedical and Health Sciences, Hiroshima University, 1-2-3 Kasumi, Minami-ku, Hiroshima 734-8553, Japan; 2 School of Pharmaceutical Sciences, Hiroshima University, 1-2-3 Kasumi, Minami-ku, Hiroshima 734-8553, Japan

**Keywords:** MT: RNA/DNA Editing, CRISPR-Cas9, genome editing, homology-directed repair, off-target effects, protein degradation

## Abstract

In genome editing, it is important to avoid off-target mutations so as to reduce unexpected side effects, especially for therapeutic applications. Recently, several high-fidelity versions of SpCas9 have been developed to reduce off-target mutations. In addition to reducing off-target effects, highly efficient intended target gene correction is also essential to rescue protein functions that have been disrupted by single nucleotide polymorphisms. Homology-directed repair (HDR) corrects genes precisely using a DNA template. Our recent development of cell cycle-dependent genome editing has shown that regulation of Cas9 activation with an anti-CRISPR-Cdt1 fusion protein increases HDR efficiency and reduces off-target effects. In this study, to apply high-fidelity SpCas9 variants to cell cycle-dependent genome editing, we evaluated anti-CRISPR inhibition of high-fidelity SpCas9s. In addition, HDR efficiency of high-fidelity SpCas9s was addressed, identifying eSpCas9, SpCas9-HF1, and LZ3 Cas9 as promising candidates. Although eSpCas9 and LZ3 Cas9 showed decreased HDR efficiency in cell cycle-dependent genome editing, SpCas9-HF1 successfully achieved increased HDR efficiency and few off-target effects when co-expressed with an AcrIIA4-Cdt1 fusion.

## Graphical abstract

**Figure:**
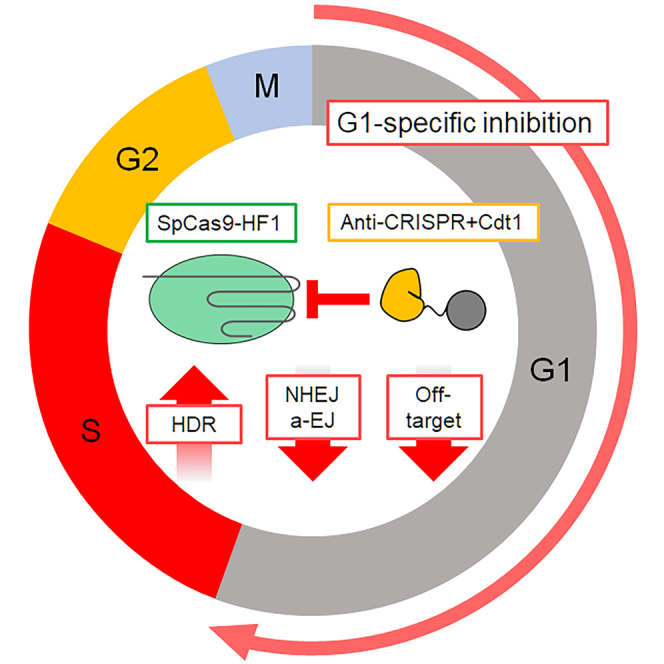

## Teaser

Nomura and colleagues applied high-fidelity SpCas9 variants to cell cycle-dependent genome editing utilizing Cdt1-fused anti-CRISPRs. It was found that several variants show higher HDR efficiency than wild-type SpCas9 by themselves, and SpCas9 HF-1 was confirmed as the best candidate showing further reduction of off-target effects in addition to increased HDR efficiency.

## Introduction

Modification of target genes in various organisms is useful not only to examine gene functions, but also to endow cells with selected functions. CRISPR-Cas9 is the most widely used tool for gene modification.^1^^,^^2^^,^^3^^,^^4^ It is important to edit target gene sequences precisely, without introducing mutations at off-target sites. Precise editing is achieved by increasing the editing ratio using homology-directed repair^5^ (HDR). HDR is a DNA repair pathway mediated by gene-repair proteins such as CtIP and the MRN complex.^6^ A customized repair template such as a donor plasmid or a single-stranded donor oligonucleotide (ssODN) is required for HDR to change nucleotide sequences. HDR acts during the S/G2 phases in the cell cycle.^7^^,^^8^ Previously, our group showed that cell cycle-dependent regulation of SpCas9 with Cdt1-fused anti-CRISPR increases HDR efficiency.^9^ In this system, an anti-CRISPR called AcrIIA4, derived from *Listeria monocytogenes* prophage^10^ is fused with amino acid residues 30 to 120 of Cdt1 (Cdt1[30–120]) to achieve cell cycle-specific inhibition of SpCas9 activity at G1 phase. In S/G2 phases, AcrIIA4-Cdt1 is degraded via ubiquitination of its Cdt1 domain, and SpCas9 is activated.

Recently, we applied AcrIIA5 from *Streptococcus thermophilus* phage D4276^11^ to cell cycle-dependent genome editing. AcrIIA5-Cdt1 increased HDR efficiency and decreased off-target effects, depending on the target DNA sequence.^12^ Moreover, Cdt1-fused anti-CRISPRs have shown synergistic increases of HDR efficiency when used with SpCas9 fused with Geminin 1-110 fragment (SpCas9-Gem), which is degraded in G1 phases.^12^^,^^13^^,^^14^ Cell cycle-dependent genome editing not only increases HDR efficiency, but also suppresses off-target effects.

We have shown that cell cycle-dependent genome editing using Cdt1-fused anti-CRISPRs is effective when combined with modified SpCas9, suggesting that it could be possible to use high-fidelity SpCas9 variants to increase precision of genome editing. Several mutants such as eSpCas9, SpCas9-HF1, HypaCas9, and R63A/Q768A Cas9 have been rationally engineered from parental SpCas9 to reduce the binding affinity of the Cas9-single guide RNA (sgRNA) complex to DNA, suppressing off-target activity.^15^^,^^16^^,^^17^^,^^18^ By site-directed mutagenesis, changing amino acid residues that interact with DNA into alanine, binding affinity of these mutants to DNA is reduced. As a result, stability of SpCas9 variants and the sgRNA complex diminishes when they bind to off-target DNA sequences, and off-target effects are thereby suppressed, although target gene editing efficiency is almost equal to that of parental SpCas9. Another approach for obtaining highly specific Cas9 variants is based on directed evolution. xCas9 is another high-fidelity variant of SpCas9, which was developed using phage-assisted continuous evolution.^19^ Among evolved xCas9 variants, xCas9(3.7) showed the best target specificity with broad PAM recognition and fewer unintended mutations. SpartaCas9 was also evolved with phage selection by introducing mutations using scanning mutagenesis of oligo-directed targets (SMOOT).^20^ An evoCas9 was evolved from SpCas9 by adding random mutations in the REC3 domain and screening in yeast.^21^ HiFi Cas9 and SniperCas9 were evolved in *E. coli* by introducing target gene sequences in the human genome into the *E. coli* genome and screening with plasmid DNA, which encodes an antibiotic resistance gene and an off-target DNA sequence.^22^^,^^23^ An LZ3 Cas9 is screened in HEK293FT cells with tagmentation-based tag integration site sequencing.^24^ In this study, we checked HDR activity of 10 high-fidelity Cas9 variants, eSpCas9, SpCas9-HF1, HiFi Cas9, HypaCas9, xCas9(3.7), evoCas9, Sniper-Cas9, LZ3 Cas9, R63A/Q768A Cas9, and SpartaCas9 at the EMX1 site and selected optimal Cas9 variants to apply to cell cycle-dependent genome editing to improve precision.

## Results

### Inhibition of SpCas9 variant activity by AcrIIA4 and AcrIIA5

First, inhibition activity of AcrIIA4 and AcrIIA5 against engineered or evolved SpCas9 variants, eSpCas9, SpCas9-HF1, HiFi Cas9, HypaCas9, and xCas9(3.7), was compared with that of parental SpCas9 (Figure S1A). As the second screening, inhibition activity of AcrIIA4 and AcrIIA5 against evoCas9, Sniper-Cas9, LZ3 Cas9, R63A/Q768A Cas9, and SpartaCas9 was also assessed (Figure S1B). Episomal expression vectors encoding genes of anti-CRISPR and SpCas9 variants sectioned by the T2A self-cleaving peptide sequence were constructed to obtain equal amounts of anti-CRISPR and SpCas9 variants in cells. Genome editing efficiency was evaluated as follows. Briefly, after transfection with vectors, 293A cells were selected with hygromycin for 3 days. Then cells were transfected with an sgRNA coding vector targeting the *EMX1* gene by lipofection. Genomic DNA was extracted 48 h after the second transfection. Target mutation efficiency was calculated using the Tracking of Indels by Decomposition (TIDE) program (https://tide.nki.nl/).^25^ Target mutation efficiency of each SpCas9 variant was almost the same as that of parental SpCas9 and both AcrIIA4 and AcrIIA5 efficiently inhibited SpCas9 variants.

### Comparison of HDR and on/off-target efficiencies of SpCas9 variants in the presence of ssODN

**Figure 1:**
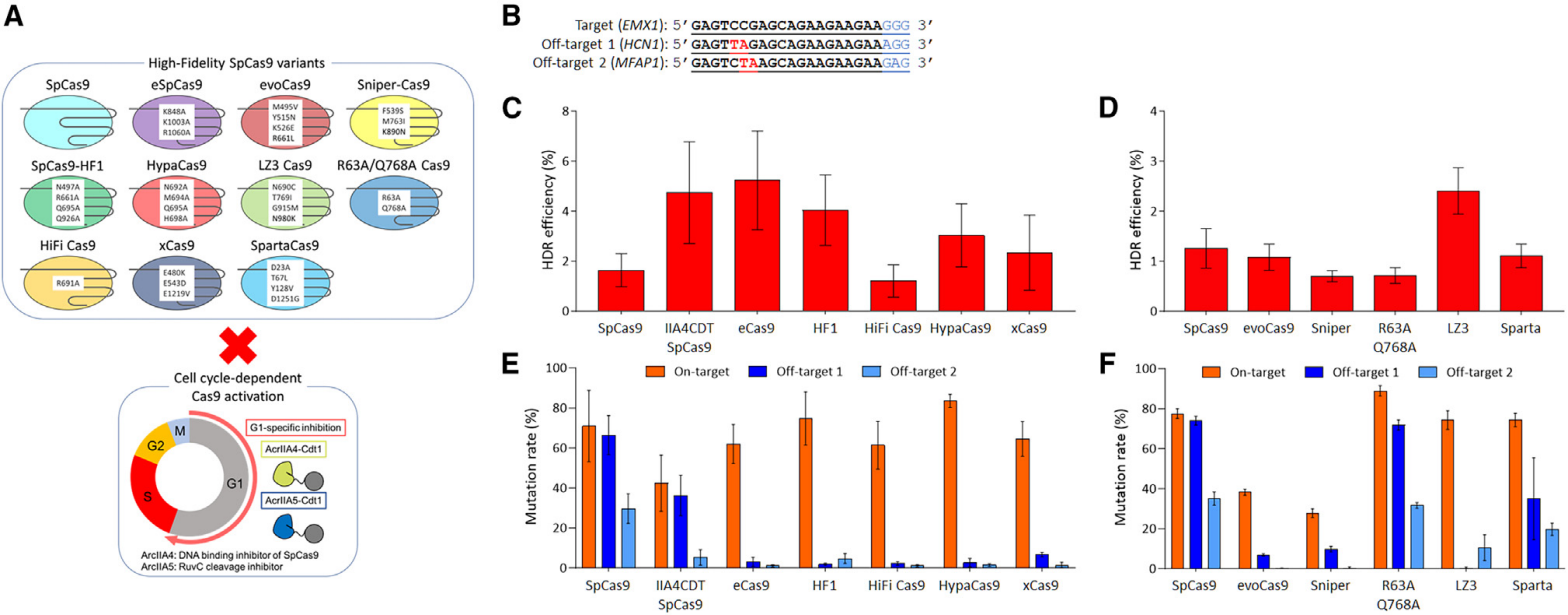
Comparison of HDR efficiency and on- and off-target mutation rates among SpCas9, AcrIIA4-Cdt1-T2A-SpCas9, and SpCas9 variants at the EMX1 site (A) Schematic representation of this study. Synergistic, precise genome editing by combining high-fidelity SpCas9 variants and anti-CRISPR mediated activity control. (B) On- and off-target sequences used in experiments. (C and D) Evaluations of HDR efficiency in the first (C) and the second (D) screenings. (E and F) Evaluations of on- and off-target mutation rates in the first (E) and the second (F) screenings. HDR efficiency was evaluated using HindIII digestion for PCR products amplified from target sites in extracted genomic DNA. HDR efficiency was calculated using the following formula: 100 × ((b + c)/(a + b + c)), where “a” is the integrated intensity of undigested PCR product, and “b” and “c” are integrated intensities of each cleavage product. On- and off-target mutation rates were analyzed by TIDE, utilizing Sanger sequencing data of PCR products amplified from target sites in extracted genomic DNA. On-target mutations include mutagenesis by a-EJ and NHEJ. Samples are identified by names under graph bars. IIA4CDTeSpCas9 means co-expression of AcrIIA4-Cdt1 and eSpCas9. n = 4 except for xCas9(3.7) off-target 1 mutation sample. n = 3 for xCas9(3.7) off-target 1 mutation sample. Significance of differences was tested with Student’s t test by comparing each sample independently with SpCas9. p values are in Table S1.

As there is relatively little information about HDR efficiency of high-fidelity SpCas9 variants, genome editing efficiency, including on/off-target mutations, was evaluated using ssODN as the donor DNA. In these experiments, selection with hygromycin after the first transfection was continued for 4–5 days, and the sgRNA encoding vector and ssODNs having an HindIII site were introduced into selected cells by electroporation. HDR efficiency was assessed by HindIII digestion of PCR-amplified fragments from the EMX-1 target site (Figure 1B). Interestingly, eSpCas9 and SpCas9-HF1 showed more than 2-fold higher HDR activity than parental SpCas9 (Figure 1C). LZ3 Cas9 also showed higher HDR activity compared with SpCas9 in the second screening (Figure 1D). HDR efficiency was almost the same as that of cell cycle-dependent activation of SpCas9 using AcrIIA4-Cdt1 in eCas9 and SpCas9-HF1. Other SpCas9 variants, HiFi Cas9, HypaCas9, xCas9(3.7), evoCas9, Sniper-Cas9, R63A/Q768A Cas9, and SpartaCas9 showed similar or lower HDR efficiency than SpCas9. There was no difference in on-target mutation efficiency among parental SpCas9 and high-fidelity SpCas9 variants (Figure 1E). All SpCas9 variants reduced off-target mutations to less than 20% compared with parental SpCas9, as previously reported (Figure 1E). Cell cycle-dependent activation of SpCas9 using AcrIIA4-Cdt1 also reduces off-target effects, but high-fidelity SpCas9 variants (except for R63A/Q768A Cas9 and SpartaCas9) reduced off-target effects more strongly (Figures 1E and 1F). Mutation positions of eSpCas9 are K848A, K1003A, and R1060A at RuvC and HNH nuclease domains.^15^ For SpCas9-HF1, mutations, N497A, R661A, Q695A, and Q926A, are introduced into the REC1 and RuvC nuclease domains.^14^ LZ3 Cas9 has an N690C mutation in REC III and T769I, G915M, and N980K mutations in the RuvC nuclease domain. These mutations reduce interactions between SpCas9 variants and target DNA strands because tertiary complexes of SpCas9 variants, sgRNA, and DNA are destabilized. In eSpCas9, it may be that a weakened interaction between eSpCas9 and the non-target DNA strand affects the location of the cleavage site by RuvC. Although the reason that HDR efficiency is increased by eSpCas9, SpCas9-HF1, and LZ3 Cas9 remains unclear, these results support the hypothesis that a combination of cell cycle-dependent activation and eSpCas9, SpCas9-HF1, or LZ3 Cas9 may synergistically increase HDR and reduce off-target effects.

### Increasing the HDR ratio by combining AcrIIA4-Cdt1 with SpCas9-HF1

**Figure 2:**
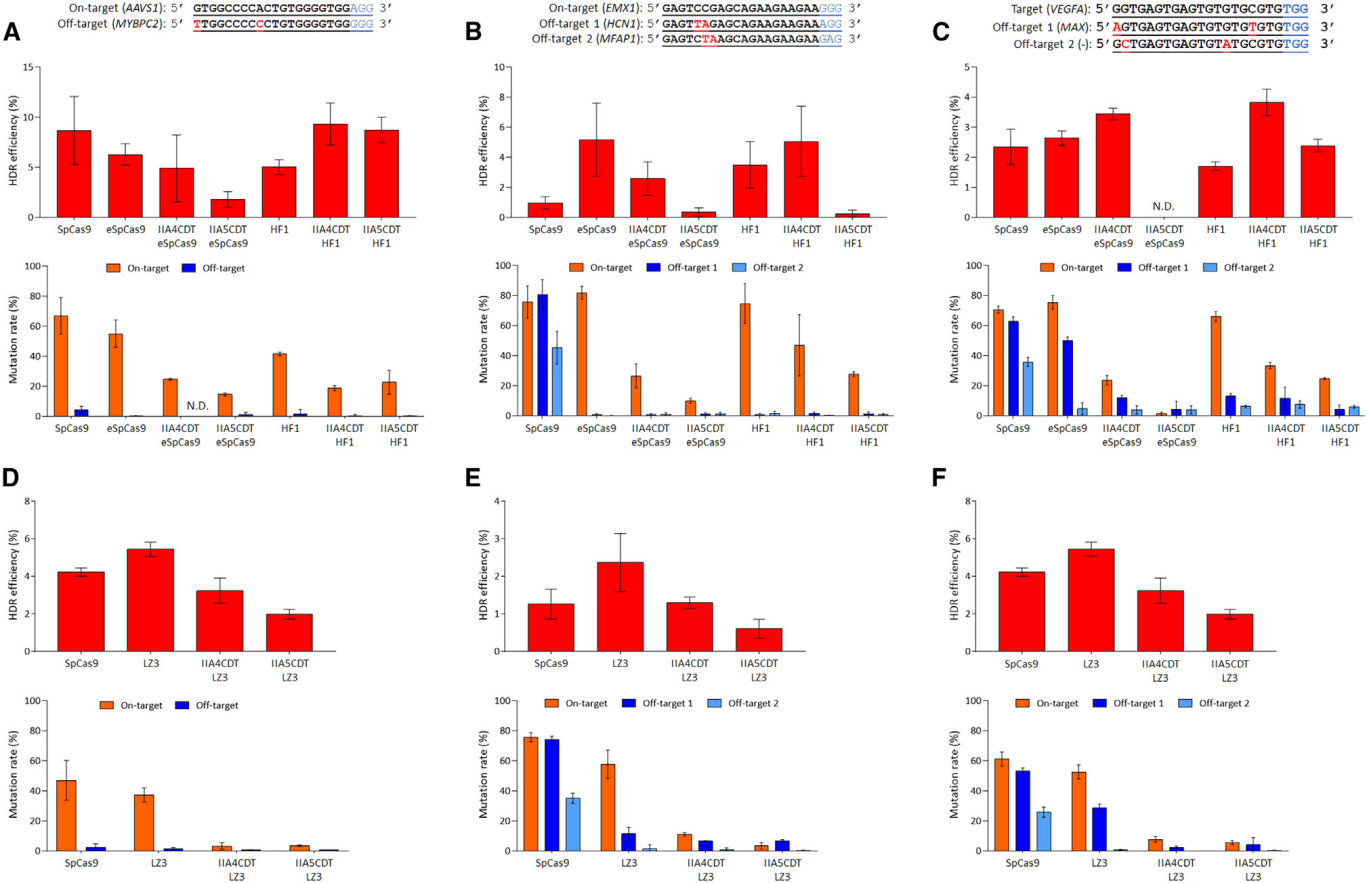
Comparison of HDR efficiency and on- and off-target mutation rates among SpCas9, eSpCas9, AcrIIA4-Cdt1-T2A-eSpCas9, AcrIIA5-Cdt1-T2A-eSpCas9, SpCas9-HF1, AcrIIA4-Cdt1-T2A-SpCas9-HF1, AcrIIA5-Cdt1-T2A-SpCas9-HF1, LZ3 Cas9, AcrIIA4-Cdt1-T2A-LZ3 Cas9, and AcrIIA5-Cdt1-T2A LZ3 Cas9 at the AAVS1 (A and D), EMX1 (B and E), and VEGFA (C and F) sites. Samples are identified by names under graph bars. IIA4CDT or IIA5CDT and SpCas9 variant names mean co-expression of AcrIIA4-Cdt1 or AcrIIA5-Cdt1 with indicated SpCas9 variants. HDR efficiency was evaluated using HindIII digestion for PCR products amplified from target sites in extracted genomic DNA. HDR efficiency was calculated with the following formula: 100 × ((b + c)/(a + b + c)), where “a” is the integrated intensity of undigested PCR product, and “b” and “c” are integrated intensities of each cleavage product. On- and off-target mutation rates were analyzed with TIDE utilizing Sanger sequencing data of PCR products amplified from target sites in extracted genomic DNA. On-target mutations include mutagenesis by a-EJ and NHEJ. n = 4 for AAVS1 and EMX1 target samples. n = 3 for VEGFA target samples. N.D., not determined. Significance in difference was tested by Student’s t test by comparing each sample independently with SpCas9. p values are in Table S2.

As the next step, effects of co-expression of AcrIIA4-Cdt1 and AcrIIA5-Cdt1 with eSpCas9, SpCas9-HF1, or LZ3 Cas9 on HDR efficiency and off-target effects were addressed. Episomal expression vectors were constructed as described above. HindIII digestion for evaluation of HDR efficiency and Sanger sequencing and TIDE analysis for evaluation of on-target mutations were performed utilizing three target sites, AAVS1, EMX1, and VEGFA. At the AAVS1 target site, increased HDR efficiency was not observed for eSpCas9, SpCas9-HF1, and LZ3 Cas9 (Figures 2A and 2D). In addition, when eSpCas9 was used with anti-CRISPR+Cdt1, HDR efficiency was decreased, especially with AcrIIA5+Cdt1, compared with SpCas9. The eSpCas9 on-target mutation rate was decreased in combination with AcrIIA4-Cdt1 or AcrIIA5-Cdt1 as well. SpCas9-HF1 showed increased HDR efficiency with AcrIIA4-Cdt1 (1.86-fold) or AcrIIA5-Cdt1 (1.74-fold) compared with SpCas9-HF1 alone, although it was almost the same as that of SpCas9. Decreased off-target effects were observed for all samples, eSpCas9 or SpCas9-HF1 alone, and those combined with AcrIIA4-Cdt1 and AcrIIA5-Cdt1, compared with SpCas9. The accuracy of targeted gene correction, calculated by dividing HDR efficiency by the on-target mutation rate, increased up to 3.77-fold for combinations of AcrIIA4-Cdt1 and SpCas9-HF1, compared with SpCas9 (Table 1).

**Table 1:** Comparison of precision while using AAVS1, EMX1, and VEGFA target sgRNA among SpCas9, eSpCas9, AcrIIA4-Cdt1-T2A-eSpCas9, AcrIIA5-Cdt1-T2A-eSpCas9, SpCas9-HF1, AcrIIA4-Cdt1-T2A-SpCas9-HF1, AcrIIA5-Cdt1-T2A-SpCas9-HF1, LZ3 Cas9, AcrIIA4-Cdt1-T2A-LZ3 Cas9, and AcrIIA5-Cdt1-T2A-LZ3 Cas9

Editing precision (HDR/on-target mutation ratio)	AAVS1 target	EMX1 target	VEGFA target
SpCas9	0.13 ± 0.03	0.01 ± 0.004	0.03 ± 0.01
eSpCas9	0.12 ± 0.03	0.07 ± 0.03	0.04 ± 0.01
AcrIIA4-Cdt1-T2A-eSpCas9	0.20 ± 0.14	0.11 ± 0.03	0.15 ± 0.03
AcrIIA5-Cdt1-T2A-eSpCas9	0.12 ± 0.04	0.03 ± 0.04	N.D.
SpCas9-HF1	0.12 ± 0.02	0.05 ± 0.01	0.03 ± 0.003
AcrIIA4-Cdt1-T2A-SpCas9-HF1	0.49 ± 0.09	0.15 ± 0.08	0.12 ± 0.02
AcrIIA5-Cdt1-T2A-SpCas9-HF1	0.43 ± 0.20	0.01 ± 0.01	0.10 ± 0.01
LZ3 Cas9	0.08 ± 0.02	0.04 ± 0.01	0.10 ± 0.004
AcrIIA4-Cdt1-T2A-LZ3 Cas9	0.23 ± 0.04	0.12 ± 0.01	0.46 ± 0.22
AcrIIA5-Cdt1-T2A-LZ3 Cas9	0.30 ± 0.06	0.30 ± 0.28	0.37 ± 0.09

N.D., not determined.

At the EMX1 site, increased HDR efficiency was observed for eSpCas9, SpCas9-HF1, and LZ3 Cas9 alone, as observed in other experiments (Figures 2B and 2E). However, eSpCas9 did not improve HDR efficiency when combined with AcrIIA4-Cdt1 or AcrIIA5-Cdt1. For SpCas9-HF1, it showed a 1.55-fold increase of HDR efficiency when used with AcrIIA4-Cdt1, compared with SpCas9-HF1 alone, although this improvement was not observed when used with AcrIIA5-Cdt1. In the case of LZ3 Cas9, AcrIIA4-Cdt1 and AcrIIA5-Cdt1 did not improve the HDR ratio as eSpCas9 did. Numerous off-target effects were observed at the EMX1 site with SpCas9, but a significant decrease was shown for all samples with eSpCas9 or SpCas9-HF1 alone and those combined with AcrIIA4-Cdt1 or AcrIIA5-Cdt1. In addition, the on-target mutation rate was suppressed when eSpCas9 or SpCas9-HF1 was used in combination with AcrIIA4-Cdt1 or AcrIIA5-Cdt1.

Finally, at the VEGFA site, eSpCas9 or SpCas9-HF1 alone did not improve HDR efficiency, but when combined with AcrIIA4-Cdt1, they improved it by 1.3-fold and 2.2-fold, respectively (Figure 2C). On the other hand, LZ3 Cas9 alone showed improvement of HDR efficiency at the VEGFA site, but enhancement of HDR efficiency was not observed when combining AcrIIA4-Cdt1 (Figure 2F). In the AcrIIA5-Cdt1 combination, eSpCas9 and LZ3 Cas9 significantly reduced HDR efficiency, while SpCas9-HF1 increased it 1.4-fold. Reduction of on-target mutation rates was observed for eSpCas9, SpCas9-HF1, or LZ3 Cas9 when they were combined with AcrIIA4-Cdt1 or AcrIIA5-Cdt1 as other sites. For off-target effects, eSpCas9 alone showed a 50% mutation rate for off-target 1, but it was significantly reduced when combined with AcrIIA4-Cdt1. This tendency was also seen with LZ3 Cas9, and for off-target 1. Off-target effects were further reduced when eSpCas9 and SpCas9-HF1 were combined with AcrIIA5-Cdt1. Results were evaluated by HDR efficiency, on-target mutation rate, and off-target mutation rate for three target sites. Sequence dependence was observed in part, but the important finding is that on-target mutation rates were reduced by a combination of high-fidelity SpCas9s and anti-CRISPR+Cdt1, which was not observed when SpCas9 was combined with anti-CRISPR+Cdt1.^9^ The reduction of on-target mutation may indicate that these high-fidelity SpCas9s are effectively suppressed by anti-CRISPR+Cdt1 during G1 phase. A combination of SpCas9-HF1 and AcrIIA4-Cdt1 increases HDR efficiency, but eSpCas9 and LZ3 Cas9 does not.

## Discussion

SpCas9 cleaves the target DNA sequence three-base from the PAM and the DNA end is blunt-ended^26^ or staggered by 1 base pair (bp).^1^^,^^27^ Ku70, which is a component of the Ku70-Ku80 heterodimer that works as a non-homologous DNA end joining (NHEJ) mediator, binds more poorly to single-stranded DNA (ssDNA) overhangs than to blunt ends in yeast.^28^ The preferential binding of Ku protein to double-stranded DNA (dsDNA) may lead to NHEJ-mediated repair by SpCas9 after cleavage. Although eSpCas9, SpCas9-HF1, and LZ3 Cas9 showed increased HDR efficiency on their own, they should not be able to escape the NHEJ repair pathway by making a long overhang dsDNA at the cleavage site, like the 30-bp ssDNA 3′ overhung dsDNA used in Ku-DNA binding experiments. Thus, involvement of other factors in the mechanism should be considered. As shown in our recent report using AcrIIA5-Cdt1 with SpCas9,^12^ we also observed that AcrIIA4-Cdt1 with SpCas9-HF1 increased HDR efficiency at all target sites and that AcrIIA5-Cdt1 with SpCas9-HF1 failed at the EMX1 site. Success of SpCas9 activity control by AcrIIA5-Cdt1 may depend on target sequences, especially in relation to their melting temperatures (Tm). Calculation of GC content (%) and Tm values (°C) of target sites with SnapGene software found 75% GC and 66°C for the AAVS1 site, 50% GC and 55°C for the EMX1 site, and 60% GC and 60°C for the VEGFA site. These results suggest that AcrIIA5-Cdt1 shows cell cycle-dependent control of SpCas9 or its variants when the Tm of a target sequence is high (about 65°C) and results in increased HDR efficiency. In contrast, when the Tm of a target sequence is relatively low, AcrIIA5-Cdt1 only inhibits SpCas9 or its variants. This may be because AcrIIA5 inhibits SpCas9 by binding to the ternary complex of target DNA-Cas9-sgRNA, and not to the Cas9-sgRNA complex like AcrIIA4. When the Tm of the target sequence is high, the SpCas9-sgRNA-DNA complex may be stabilized, cleaving the target DNA efficiently. Above all, AcrIIA4-Cdt1, as an excellent modulator of SpCas9 or SpCas9-HF1, can increase HDR efficiency without target sequence bias, which may result from inhibiting Cas9 DNA binding, but AcrIIA5-Cdt1 is superior to AcrIIA4-Cdt1 in that it has fewer off-target effects.

eSpCas9, SpCas9-HF1, and LZ3 Cas9 increased HDR efficiency on their own, but only SpCas9-HF1 increased HDR efficiency when combined with an anti-CRISPR+Cdt1 fusion. This may be because the mutation points in eSpCas9 and LZ3 Cas9 affect the interaction of the eSpCas9/LZ3 Cas9-sgRNA complex with anti-CRISPRs. The protein expression level of eSpCas9 and SpCas9-HF1 was not changed even when co-expressed with AcrIIA4-Cdt1 or AcrIIA5-Cdt1 in the presence or absence of sgRNA (Figure S2). This supports the conclusion that reduced HDR efficiency of eSpCas9 with anti-CRISPR+Cdt1 is not caused by eSpCas9 degradation as a result of binding ubiquitinated Cdt1. The crystal structure of SpCas9-sgRNA in complex with AcrIIA4 suggests that AcrIIA4 interacts with the RuvC-III, Topo, and CTD domains of SpCas9.^29^ In comparing mutated residues of eSpCas9 and SpCas9-HF1, the K1003A and R1060A mutations of eSpCas9 are in the RuvC-III domain, but only one Q926A mutation of SpCas9-HF1 is. LZ3 Cas9 also has G915M and N980K mutations in the RuvC-III domain. Thus, these mutations in eSpCas9 and LZ3 Cas9 may affect their interaction with AcrIIA4, resulting in different HDR efficiency between eSpCas9 and SpCas9-HF1. AcrIIA5 has been reported as a broad-spectrum inhibitor against Type II Cas9.^30^ The detailed mechanism of inhibition by AcrIIA5 has not yet been identified, but it has been suggested that the N-terminal intrinsically disordered region is a critical domain for inhibition.^5^ Further investigation into the structure and inhibition mechanism of AcrIIA5 is needed to explain why there is a difference in HDR efficiency, depending on target sequences, when using AcrIIA5-Cdt1 with eSpCas9, SpCas9-HF1, or LZ3 Cas9.

## Materials and methods

### Plasmid construction

pEBMulti-Hyg was purchased from FUJIFILM. The gRNA Cloning Vector and SpCas9 were gifts from George Church (Addgene plasmids # 41824 and 41815). eSpCas9(1.1) was a gift from Feng Zhang (Addgene plasmid # 71814). A BPK4410 - human expression plasmid for SpCas9 Cluster 1 (HypaCas9) was a gift from Jennifer Doudna and Keith Joung (Addgene plasmid # 101178). xCas9(3.7) was a gift from David Liu (Addgene plasmid # 108379). A HiFi Cas9 was constructed from a SpCas9 plasmid by introducing an R691A mutation with primers, HiFi mut R691A Fw, and HiFi mut R691A Rv. Plasmids for evoCas9, Sniper-Cas9, LZ3 Cas9, and R63A/Q768A Cas9 expression were constructed by introducing mutations indicated in Figure 1A to the SpCas9 plasmid. Each SpCas9 variant gene was amplified with KOD one polymerase (TOYOBO) and introduced into NotI digested pEB vector using the Gibson Assembly Master Mix (NEB). Assembled plasmids were transformed into NEB stable competent cells (NEB). For construction of AcrIIA4/5-Cdt1-T2A-SpCas9 variants, AcrIIA4-Cdt1 and AcrIIA5-Cdt1 fragments were amplified from pEB.AcrIIA4-Cdt1-T2A-SpCas9 and pEB.AcrIIA5-Cdt1-T2A-SpCas9.^8^^,^^11^ AcrIIA4-Cdt1 or AcrIIA5-Cdt1 fragment was assembled with each SpCas9 variant and introduced into the pEB vector by Gibson Assembly.

### Cell culture and transfection

293A cells (Invitrogen) were maintained in DMEM supplemented with 10% fetal bovine serum and penicillin/streptomycin at 37°C in an atmosphere of 5% CO_2_. After introduction of the episomal vector encoding SpCas9 variants, AcrIIA4-Cdt1-T2A-SpCas9 variants, or AcrIIA5-Cdt1-T2A-SpCas9 variants using Lipofectamine 3000 (Thermo Fisher Scientific), cells were selected using 350 μg/mL Hygromycin solution (FUJIFILM) for 3 days when lipofection was used for introduction of sgRNA plasmid and for 4–5 days when electroporation was used for introduction of sgRNA plasmid and ssODN. A Neon Transfection System 10 μL Kit (Thermo Fisher Scientific) was used for electroporation. In the HDR assessment using ssODN as the template, 50 pmol of ssODN and 250 ng of sgRNA plasmid were used for 1 × 10^5^ cells and conditions for electroporation were as follows: a pulse voltage of 1,245 V, pulse width of 10 ms, and three pulses.

### Restriction enzyme assay and TIDE analysis

Forty-eight hours after transfection, genomic DNA was extracted using a QIAamp DNA Mini Kit (Qiagen). Genomic DNA (100 ng) was amplified using Tks Gflex DNA polymerase (TaKaRa) with T7E1 primers of each target. PCR conditions were 94°C for 2 min for the first denaturation, pre-amplification using 10 cycles of 98°C for 10 s, 68°C–63.5°C (−0.5°C per a cycle) for 15 s, and 68°C for 30 s, amplification using 25 cycles of 98°C for 10 s, 63°C for 15 s, and 68°C for 30 s, and a final extension at 68°C for 1 min. PCR-fragmented DNA was purified using a QIAquick PCR Purification Kit (Qiagen). Fragmented DNA (100 ng) was prepared for Sanger sequencing with a primer, and sequencing was performed by Eurofins Genomics. Sequence data were analyzed with TIDE.^25^

In the restriction enzyme assay, 200 ng of amplified DNA was reacted with 0.5 μL of HindIII (NEB) in Cutsmart buffer (NEB) at 37°C for 1 h. After the reaction, HindIII was inactivated at 65°C for 20 min. Reacted samples were analyzed using MultiNA. The indel efficiency of indel was calculated as:

100∗((a+b)/(a+b+c)),

where a and b represent areas of cleaved fragments and c represents the area of an uncleaved fragment.

## Data and code availability

Data that support the findings of this study are available in the supplemental information.

## Supplemental information

Document S1. Figures S1 and S2 and Tables S1‒S3

Document S2. Article plus supplemental information
