# Supplementary material for: SpCas9-HF1 enhances accuracy of cell cycle-dependent genome editing by increasing HDR efficiency, and by reducing off-target effects and indel rates
Source: Mol Ther Nucleic Acids. 2024 Jan 23;35(1):102124. doi: 10.1016/j.omtn.2024.102124 (PMC10848011; doi:10.1016/j.omtn.2024.102124)
Supplement: Document S1. Figures S1 and S2 and Tables S1‒S3 [file mmc1.pdf]

OMTN, Volume *35*

Supplemental information

SpCas9-HF1 enhances accuracy of cell cycle-dependent genome editing by increasing HDR efficiency, and by reducing off-target effects and indel rates

Daisuke Matsumoto, Erina Matsugi, Kanae Kishi, Yuto Inoue, Kiyomi Nigorikawa, and Wataru Nomura

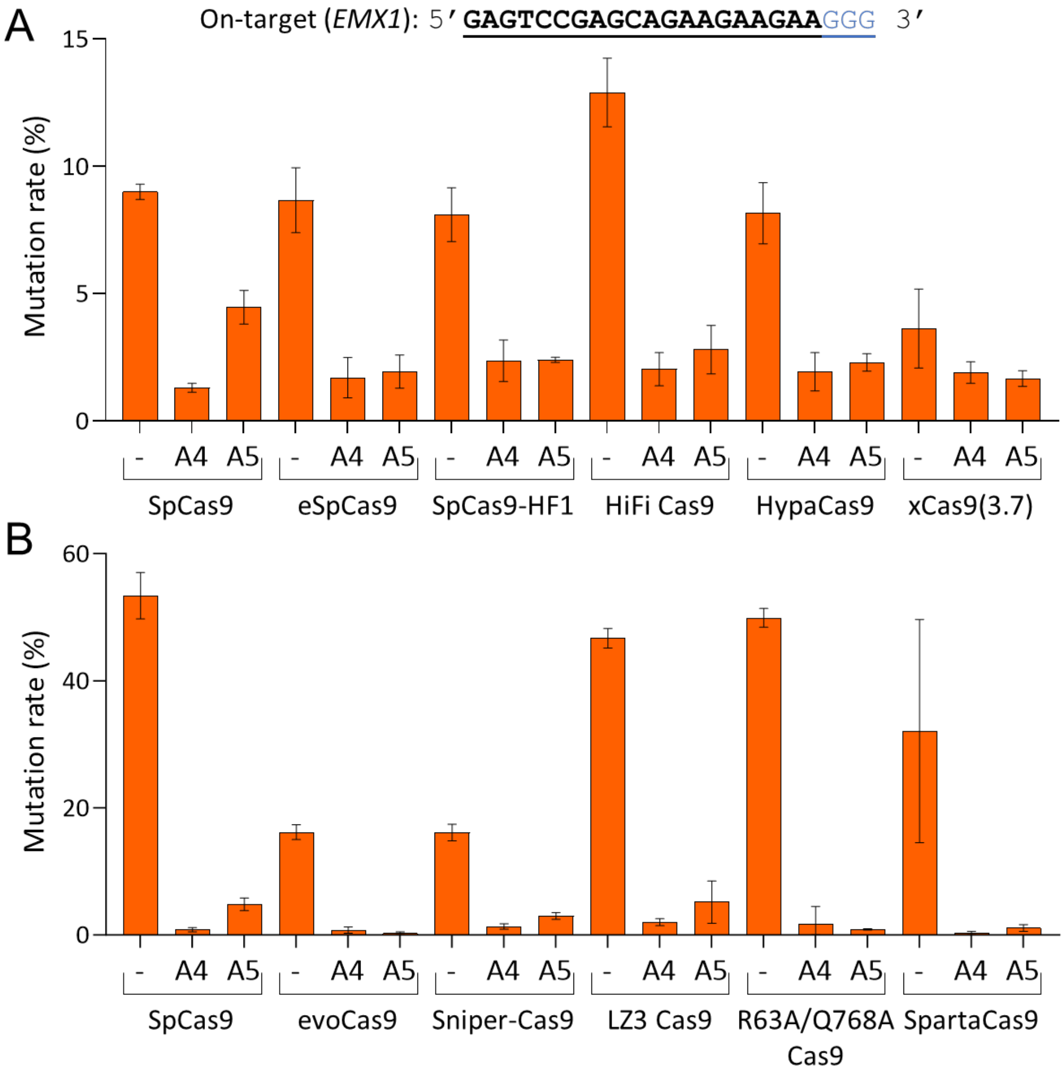

Figure S1. Evaluation of inhibition of Cas9 variants by AcrIIA4 and AcrIIA5. Mutation rates for Cas9 variants utilized in the first (A) and the second (B) screenings. The on-target DNA sequence (EMX1 site) is shown above the graph. Each PCR product amplified from extracted genomic DNA was sequenced and analyzed using TIDE. Graph bars show absence of anti-CRISPR (-) or in presence of AcrIIA4 (A4) and AcrIIA5 (A5). $n = 3$.

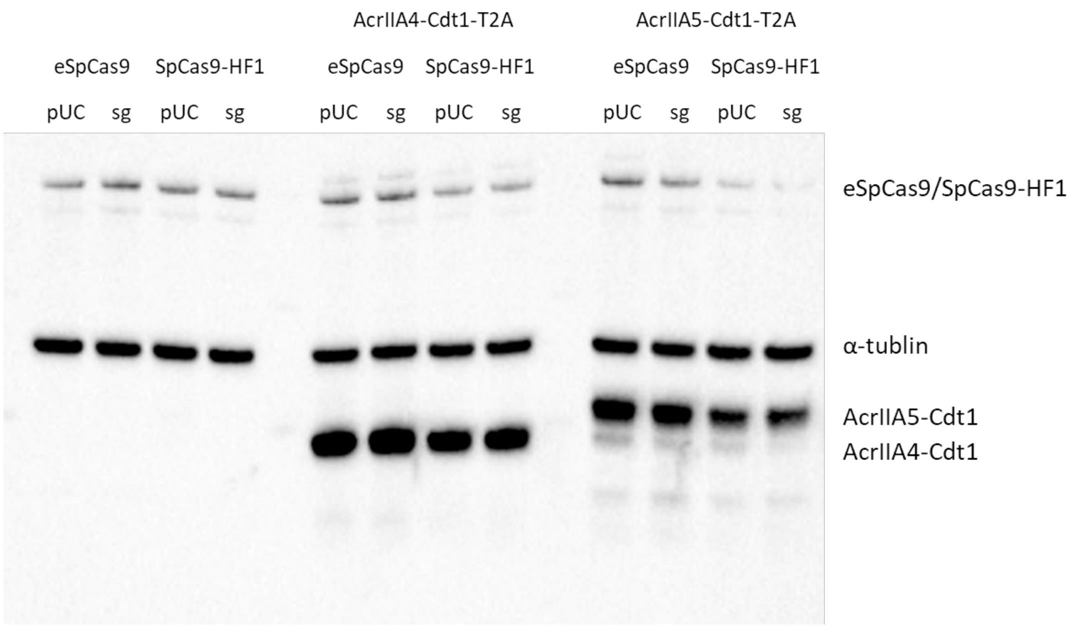

Figure S2. Protein expression levels of eSpCas9, SpCas9-HF1, AcrIIA4-Cdt1, and AcrIIA5-Cdt1 in the presence (sg) or absence (pUC) of sgRNA.

Table S1. All data used in Figure 1. Identical sample names indicate that data were obtained from the same cell population.

HDR	SpCas9	AcrIIA4-Cdt1-T2A-SpCas9	eSpCas9	SpCas9-HF1	HiFi Cas9	HypaCas9	xCas9(3.7)
Sample #1	0.75	3.17	4.20	5.65	0.71	1.19	1.34
Sample #2	1.56	2.91	3.06	4.77	0.64	3.60	1.22
Sample #3	2.25	7.10	7.47	2.70	1.47	4.02	4.46
Sample #4	2.01	5.80	6.19	3.03	2.00	3.32	2.34
Average	1.64	4.75	5.23	4.04	1.21	3.03	2.34
Standard deviation	0.66	2.04	1.98	1.41	0.65	1.26	1.50
p-value compared with Cas9 sample	-	0.03	0.01	0.02	0.38	0.10	0.43

On-target mutation	SpCas9	AcrIIA4-Cdt1-T2A-SpCas9	eSpCas9	SpCas9-HF1	HiFi Cas9	HypaCas9	xCas9(3.7)
Sample #1	80.9	31.5	52	84	73.5	83.1	60.4
Sample #2	59.8	38.6	55.1	87.8	46.7	83.5	73.2
Sample #3	52.4	36.4	69.6	60	68.2	87.8	54.3
Sample #4	90.7	63	70.9	67.1	57.0	79.8	69.9
Average	71.0	42.4	61.9	74.7	61.4	83.6	64.5
Standard deviation	17.9	14.1	9.74	13.3	11.9	3.28	8.67
p-value compared with Cas9 sample	-	0.046	0.408	0.746	0.406	0.215	0.537

Off-target 1 mutation	SpCas9	AcrIIA4-Cdt1-T2A-SpCas9	eCas9	HFCas9	HiFiCas9	HypaCas9	xCas9
Sample #1	79.5	26.9	1.50	0.00	1.60	1.50	5.60
Sample #2	68.4	33.9	0.80	0.00	1.90	0.50	6.60
Sample #3	58	33.6	4.90	2.7	3.60	5.40	7.80
Sample #4	59.9	50.6	5.10	2.90	2.00	3.00	-
Average	66.45	36.3	3.08	1.40	2.28	2.60	6.67
Standard deviation	9.80	10.1	2.24	1.62	0.90	2.13	1.10
p-value compared with Cas9 sample	-	5.14×10^{-3}	1.53×10^{-5}	1.22×10^{-5}	1.26×10^{-5}	1.44×10^{-5}	1.51×10^{-4}

Off-target 2 mutation	SpCas9	AcrIIA4-Cdt1-T2A-SpCas9	eCas9	HFCas9	HiFiCas9	HypaCas9	xCas9
Sample #1	37.1	1.50	1.30	0.00	0.40	0.80	0.50
Sample #2	34.6	9.20	0.50	0.00	1.30	2.00	0.90
Sample #3	25.4	2.40	1.70	2.70	0.90	2.10	0.50
Sample #4	21.5	8.00	0.80	2.90	1.70	1.00	3.60
Average	29.65	5.28	1.08	1.40	1.08	1.48	1.38
Standard deviation	7.40	3.89	0.53	1.62	0.56	0.67	1.50
p-value compared with Cas9 sample	-	1.12×10^{-3}	2.52×10^{-4}	3.00×10^{-4}	2.52×10^{-4}	2.74×10^{-4}	2.94×10^{-4}

HDR	SpCas9	evoCas9	Sniper-Cas9	R63A/Q768A Cas9	LZ3 Cas9	SpartaCas9
Sample #1	1.45	1.29	0.54	0.89	2.23	1.43
Sample #2	0.84	0.88	0.78	0.61	1.88	1.15
Sample #3	1.02	1.33	0.77	0.55	2.97	0.92
Sample #4	1.71	0.83	0.71	0.80	2.55	0.94
Average	1.26	1.08	0.70	0.71	2.41	1.11
Standard deviation	0.396	0.263	0.110	0.158	0.464	0.236
p-value compared with Cas9 sample	-	0.485	0.035	0.0436	0.00933	0.539

On-target mutation	SpCas9	evoCas9	Sniper-Cas9	R63A/Q768A Cas9	LZ3 Cas9	SpartaCas9
Sample #1	79.7	36.1	27.8	91.8	80.9	74.8
Sample #2	79	39.8	24.7	85.8	70.8	78.7
Sample #3	74.2	38.5	29.8	88	71.2	70.7
Sample #4	77.3	38.5	28.7	90	74.4	73.1
Average	77.55	38.23	27.75	88.90	74.33	74.33
Standard deviation	2.450	1.544	2.192	2.585	4.670	3.367
p-value compared with Cas9 sample	-	1.65×10^{-7}	8.58×10^{-8}	7.01×10^{-4}	0.267	0.172

Off-target 1 mutation	SpCas9	evoCas9	Sniper-Cas9	R63A/Q768A Cas9	LZ3 Cas9	SpartaCas9
Sample #1	72.3	7.1	11.4	68.2	0	49.6
Sample #2	77.2	6.4	8.9	72.6	0	42.7
Sample #3	73.9	7.5	10.5	72.6	0	43
Sample #4	72.7	6.5	8.4	74	1.1	4.7
Average	74.03	6.88	9.80	71.85	0.28	35.00
Standard deviation	2.22	0.519	1.39	2.52	0.550	20.45
p-value compared with Cas9 sample	-	1.62×10^{-9}	4.87×10^{-9}	0.243	9.42×10^{-10}	0.00903

Off-target 2 mutation	SpCas9	evoCas9	Sniper-Cas9	R63A/Q768A Cas9	LZ3 Cas9	SpartaCas9
Sample #1	33.7	0	0.2	33.6	4.7	21.8
Sample #2	39.9	0	1.1	31.8	14.9	21.9
Sample #3	32.6	0.4	0.1	30.2	17.1	15.3
Sample #4	33.9	0	0	30.9	5.2	19.8
Average	35.03	0.10	0.35	31.63	10.48	19.70
Standard deviation	3.30	0.200	0.51	1.47	6.446	3.09
p-value compared with Cas9 sample	-	7.33×10^{-7}	8.10×10^{-8}	0.109	5.03×10^{-4}	5.03×10^{-4}

Table S2. All data used in Figure 2. Identical sample names indicate that data were obtained from the same cell population. N.D., not determined.

AAVS1 site

HDR	SpCas9	eSpCas9	AcrIIA4-Cdt1-T2A-eSpCas9	AcrIIA5-Cdt1-T2A-eSpCas9	SpCas9-HF1	AcrIIA4-Cdt1-T2A-SpCas9-HF1	AcrIIA5-Cdt1-T2A-SpCas9-HF1
Sample #1	7.83	7.43	2.63	1.25	5.30	9.87	10.0
Sample #2	5.74	5.29	3.34	1.41	5.56	11.1	7.48
Sample #3	12.4	6.08	8.72	2.68	4.17	6.98	8.73
Average	8.66	6.27	4.90	1.78	5.01	9.32	8.72
Standard deviation	3.41	1.08	3.33	0.79	0.74	2.12	1.24
p-value compared with Cas9 sample	-	0.311	0.243	0.0272	0.144	0.791	0.979

On-target mutation	SpCas9	eCas9	AcrIIA4-Cdt1-T2A-eCas9	AcrIIA5-Cdt1-T2A-eCas9	HF1Cas9	AcrIIA4-Cdt1-T2A-HF1Cas9	AcrIIA5-Cdt1-T2A-HF1Cas9
Sample #1	58.8	57.6	24.4	14.5	40.3	17.9	26.5
Sample #2	61.3	62.6	25.4	13.6	41.2	20.7	27.9
Sample #3	80.9	44.9	24.1	15.7	42.8	17.6	13.5
Average	67.0	55.0	24.6	14.6	41.4	18.7	22.6
Standard deviation	12.1	9.12	0.68	1.05	1.27	1.71	7.94
p-value compared with Cas9 sample	-	0.243	0.00376	0.00172	0.0220	0.00239	0.00605

Off-target mutation	SpCas9	eCas9	AcrIIA4-Cdt1-T2A-eCas9	AcrIIA5-Cdt1-T2A-eCas9	HF1Cas9	AcrIIA4-Cdt1-T2A-HF1Cas9	AcrIIA5-Cdt1-T2A-HF1Cas9
Sample #1	6.70	0.00	0.00	2.80	0.00	0.00	0.00
Sample #2	2.30	0.00	0.00	0.30	0.00	0.00	0.00
Sample #3	4.20	0.50	0.00	0.70	5.00	1.30	0.60
Average	4.40	0.17	0.00	1.27	1.67	0.43	0.20
Standard deviation	2.21	0.29	0.00	1.34	2.89	0.75	0.35
p-value compared with Cas9 sample	-	0.0301	0.0260	0.104	0.263	0.0421	0.0312

HDR	SpCas9	LZ3 Cas9	AcrIIA4-Cdt1-T2A-LZ3 Cas9	AcrIIA5-Cdt1-T2A-LZ3 Cas9
Sample #1	4.80	4.18	1.06	1.30
Sample #2	5.73	3.24	0.05	0.94
Sample #3	5.11	2.66	0.93	0.72
Sample #4	3.07	2.20	0.92	1.21
Average	4.68	3.07	0.74	1.04
Standard deviation	1.142	0.853	0.463	0.263
p-value compared with Cas9 sample	-	0.0650	6.94×10^{-4}	8.14×10^{-4}

On-target mutation	SpCas9	LZ3 Cas9	AcrIIA4-Cdt1-T2A-LZ3 Cas9	AcrIIA5-Cdt1-T2A-LZ3 Cas9
Sample #1	55.3	37.2	5.1	4.5

Sample #2	48.3	41	0	3.1
Sample #3	56.5	40	4.5	3.1
Sample #4	27.9	30.7	3.3	3.2
Average	47.0	37.2	3.23	3.48
Standard deviation	13.2	4.64	2.28	0.685
p-value compared with Cas9 sample	-	0.213	6.22×10^{-4}	5.97×10^{-4}

Off-target mutation	SpCas9	LZ3 Cas9	AcrIIA4-Cdt1-T2A-LZ3 Cas9	AcrIIA5-Cdt1-T2A-LZ3 Cas9
Sample #1	5.50	2.00	0.70	0.50
Sample #2	2.80	0.70	0.30	0.60
Sample #3	0.40	0.80	0.80	0.60
Sample #4	0.90	2.30	0.50	0.60
Average	4.40	0.17	0.00	1.27
Standard deviation	2.21	0.29	0.00	1.34
p-value compared with Cas9 sample	-	2.31	0.819	0.222

EMX1 site

HDR	SpCas9	eSpCas9	AcrIIA4-Cdt1-T2A-eSpCas9	AcrIIA5-Cdt1-T2A-eSpCas9	SpCas9-HF1	AcrIIA4-Cdt1-T2A-SpCas9-HF1	AcrIIA5-Cdt1-T2A-SpCas9-HF1
Sample #1	0.75	6.04	3.58	0.68	5.65	5.79	0.57
Sample #2	1.56	9.12	4.03	0.63	4.77	6.47	0.40
Sample #3	1.07	2.50	1.82	0.00	2.84	5.75	0.00
Sample #4	0.98	4.96	2.08	0.00	2.22	6.05	0.00
Average	1.09	5.65	2.88	0.33	3.87	6.01	0.24
Standard deviation	0.34	2.75	1.09	0.38	1.61	0.33	0.29
p-value compared with Cas9 sample	-	0.0164	0.0203	0.0247	0.0148	8.17×10^{-7}	0.00896

On-target mutation	SpCas9	eSpCas9	AcrIIA4-Cdt1-T2A-eSpCas9	AcrIIA5-Cdt1-T2A-eSpCas9	SpCas9-HF1	AcrIIA4-Cdt1-T2A-SpCas9-HF1	AcrIIA5-Cdt1-T2A-SpCas9-HF1
Sample #1	78.9	78.5	34.8	8.2	84.0	62.3	27.8
Sample #2	83.2	88.1	29.0	11.3	87.8	64.7	28.0
Sample #3	80.9	80.9	26.1	11.0	60.0	38.6	25.6
Sample #4	59.8	79.9	16.0	8.8	67.1	22.0	29.4
Average	75.70	81.85	26.48	9.83	74.73	46.90	27.70
Standard deviation	10.74	4.28	7.86	1.55	13.32	20.35	1.57
p-value compared with Cas9 sample	-	0.328	3.14×10^{-4}	1.90×10^{-5}	0.9130	0.0464	1.16×10^{-4}

Off-target 1 mutation	SpCas9	eCas9	AcrIIA4-Cdt1-T2A-eCas9	AcrIIA5-Cdt1-T2A-eCas9	HF1Cas9	AcrIIA4-Cdt1-T2A-HF1Cas9	AcrIIA5-Cdt1-T2A-HF1Cas9
Sample #1	82.5	1.00	0.00	0.20	0.00	0.20	0.00
Sample #2	92.5	0.00	0.10	0.00	0.00	2.30	0.00
Sample #3	79.5	1.50	1.80	1.60	2.70	2.00	2.70
Sample #4	68.4	0.60	0.70	1.90	2.90	1.50	1.80
Average	80.73	0.78	0.65	0.93	1.40	1.50	1.13
Standard deviation	9.92	0.63	0.83	0.96	1.62	0.93	1.35
p-value compared with Cas9 sample	-	3.67×10^{-6}	3.66×10^{-6}	3.77×10^{-6}	4.10×10^{-6}	3.923×10^{-6}	3.92×10^{-6}

Off-target 2 mutation	SpCas9	eCas9	AcrIIA4-Cdt1-T2A-eCas9	AcrIIA5-Cdt1-T2A-eCas9	HF1Cas9	AcrIIA4-Cdt1-T2A-HF1Cas9	AcrIIA5-Cdt1-T2A-HF1Cas9
Sample #1	55.5	0.00	1.40	0.00	0.00	0.00	0.00
Sample #2	53.7	0.00	2.10	1.90	0.00	0.00	0.50
Sample #3	37.1	0.00	0.10	0.60	2.70	0.60	1.50
Sample #4	34.6	0.20	0.00	2.20	2.90	0.00	1.40
Average	45.23	0.05	0.90	1.18	1.40	0.15	0.85
Standard deviation	10.90	0.10	1.02	1.05	1.62	0.30	0.72
p-value compared with Cas9 sample	-	1.67×10^{-4}	1.90×10^{-4}	1.97×10^{-4}	2.10×10^{-4}	1.69×10^{-4}	1.87×10^{-4}

HDR	SpCas9	LZ3 Cas9	AcrIIA4-Cdt1-T2A-LZ3 Cas9	AcrIIA5-Cdt1-T2A-LZ3 Cas9
Sample #1	1.449	1.854	1.446	0.284
Sample #2	0.844	3.293	1.339	0.774
Sample #3	1.023	1.613	1.313	0.829
Sample #4	1.713	2.701	1.086	0.523
Average	1.26	2.37	1.30	0.60
Standard deviation	0.396	0.775	0.151	0.251
p-value compared with Cas9 sample	-	0.0437	0.861	0.0314

On-target mutation	SpCas9	LZ3 Cas9	AcrIIA4-Cdt1-T2A-LZ3 Cas9	AcrIIA5-Cdt1-T2A-LZ3 Cas9
Sample #1	72.3	56.4	11.5	0.40
Sample #2	79	58.1	12.5	5.00
Sample #3	74.2	46.6	10.3	4.60
Sample #4	77.3	69.6	9.90	3.50
Average	75.70	57.7	11.1	3.38
Standard deviation	3.014	9.43	1.18	2.08
p-value compared with Cas9 sample	-	0.0108	1.65×10^{-8}	1.76×10^{-8}

Off-target 1 mutation	SpCas9	LZ3 Cas9	AcrIIA4-Cdt1-T2A-LZ3 Cas9	AcrIIA5-Cdt1-T2A-LZ3 Cas9
Sample #1	72.3	17.2	6.20	6.50
Sample #2	77.2	7.60	6.80	7.90
Sample #3	73.9	12.4	6.80	6.50
Sample #4	72.7	8.50	6.20	6.20

Average	74.0	11.4	6.50	6.78
Standard deviation	2.22	4.38	0.35	0.76
p-value compared with Cas9 sample	-	2.40×10^{-7}	1.44×10^{-9}	1.91×10^{-9}

Off-target 2 mutation	SpCas9	LZ3 Cas9	AcrIIA4-Cdt1-T2A-LZ3 Cas9	AcrIIA5-Cdt1-T2A-LZ3 Cas9
Sample #1	33.7	5.50	0	0
Sample #2	39.9	0	2.70	0.70
Sample #3	32.6	0	0	0
Sample #4	33.9	0.10	0.10	0
Average	35.03	1.40	0.70	0.18
Standard deviation	3.30	2.73	1.33	0.350
p-value compared with Cas9 sample	-	4.24×10^{-6}	1.26×10^{-6}	7.59×10^{-7}

VEGFA site

HDR	SpCas9	eSpCas9	AcrIIA4-Cdt1-T2A-eSpCas9	AcrIIA5-Cdt1-T2A-eSpCas9	SpCas9-HF1	AcrIIA4-Cdt1-T2A-SpCas9-HF1	AcrIIA5-Cdt1-T2A-SpCas9-HF1
Sample #1	1.68	2.43	3.25	0.00	1.54	3.65	2.63
Sample #2	2.56	2.57	3.43	0.00	1.75	4.33	2.30
Sample #3	2.79	2.90	3.64	0.00	1.82	3.50	2.24
Average	2.35	2.63	3.44	0.00	1.70	3.83	2.39
Standard deviation	0.59	0.24	0.19	0.00	0.15	0.44	0.21
p-value compared with Cas9 sample	-	0.476	0.0377	0.00233	0.142	0.0250	0.909

On-target mutation	SpCas9	eSpCas9	AcrIIA4-Cdt1-T2A-eSpCas9	AcrIIA5-Cdt1-T2A-eSpCas9	SpCas9-HF1	AcrIIA4-Cdt1-T2A-SpCas9-HF1	AcrIIA5-Cdt1-T2A-SpCas9-HF1
Sample #1	72.7	80.3	23.1	0.90	69.8	34.8	24.6
Sample #2	71.2	74.8	26.8	2.5	64.3	30.8	25.4
Sample #3	68.2	71.4	20.6	0.70	64.0	34.4	24.1
Average	70.70	75.50	23.50	1.37	66.03	33.33	24.70
Standard deviation	2.29	4.49	3.12	0.99	3.27	2.20	0.66
p-value compared with Cas9 sample	-	0.174	2.97×10^{-5}	1.11×10^{-6}	0.113	3.44×10^{-5}	4.78×10^{-6}

Off-target 1 mutation	SpCas9	eSpCas9	AcrIIA4-Cdt1-T2A-eSpCas9	AcrIIA5-Cdt1-T2A-eSpCas9	SpCas9-HF1	AcrIIA4-Cdt1-T2A-SpCas9-HF1	AcrIIA5-Cdt1-T2A-SpCas9-HF1
Sample #1	59.2	52.7	9.90	0.50	12.0	17.9	2.70
Sample #2	64.6	48.8	13.6	1.90	15.0	13.5	2.00
Sample #3	64.3	49.0	12.0	10.5	11.8	3.50	7.60
Average	62.7	50.2	11.8	4.30	12.9	11.6	4.10
Standard deviation	3.03	2.20	1.86	5.41	1.79	7.38	3.05
p-value compared with Cas9 sample	-	0.00440	1.58×10^{-5}	8.30×10^{-5}	1.65×10^{-5}	3.77×10^{-4}	1.92×10^{-5}

Off-target 2 mutation	SpCas9	eSpCas9	AcrIIA4-Cdt1-T2A-eSpCas9	AcrIIA5-Cdt1-T2A-eSpCas9	SpCas9-HF1	AcrIIA4-Cdt1-T2A-SpCas9-HF1	AcrIIA5-Cdt1-T2A-SpCas9-HF1
Sample #1	32.9	0.00	0.70	5.70	6.70	9.90	7.00
Sample #2	38.9	7.30	5.60	5.20	6.60	7.60	5.50
Sample #3	35.3	6.40	5.30	0.70	5.20	5.00	5.40
Average	35.7	4.57	3.87	3.87	6.17	7.50	5.97
Standard deviation	3.02	3.98	2.75	2.75	0.84	2.45	0.90
p-value compared with Cas9 sample	-	4.18×10^{-4}	1.74×10^{-4}	1.75×10^{-4}	8.25×10^{-5}	2.31×10^{-4}	8.19×10^{-5}

HDR	SpCas9	LZ3 Cas9	AcrIIA4-Cdt1-T2A-LZ3 Cas9	AcrIIA5-Cdt1-T2A-LZ3 Cas9
Sample #1	3.99	5.85	2.86	2.28
Sample #2	4.43	5.35	2.82	1.83
Sample #3	4.25	5.11	4.01	1.83
Average	4.22	5.44	3.23	1.98
Standard deviation	0.223	0.376	0.676	0.257
p-value compared with Cas9 sample	-	0.00857	0.0739	3.36×10^{-4}

On-target mutation	SpCas9	LZ3 Cas9	AcrIIA4-Cdt1-T2A-LZ3 Cas9	AcrIIA5-Cdt1-T2A-LZ3 Cas9
Sample #1	65.2	56.5	9.5	6.2
Sample #2	62.4	53.7	7.8	6.4
Sample #4	56.1	47.5	5.7	4.0
Average	61.23	52.57	7.67	5.53
Standard deviation	4.66	4.61	1.90	1.33
p-value compared with Cas9 sample	-	0.0838	5.10×10^{-5}	3.76×10^{-5}

Off-target 1 mutation	SpCas9	LZ3 Cas9	AcrIIA4-Cdt1-T2A-LZ3 Cas9	AcrIIA5-Cdt1-T2A-LZ3 Cas9
Sample #1	53.3	31.4	2.8	0.6
Sample #2	51.4	28.2	1.7	9.6
Sample #3	55.2	26.4	3.0	2.4
Average	53.3	28.7	2.5	4.2
Standard deviation	1.90	2.53	0.70	4.76
p-value compared with Cas9 sample	-	1.75×10^{-4}	1.68×10^{-6}	7.74×10^{-5}

Off-target 2 mutation	SpCas9	LZ3 Cas9	AcrIIA4-Cdt1-T2A-LZ3 Cas9	AcrIIA5-Cdt1-T2A-LZ3 Cas9
Sample #1	23	0.10	0	0.50
Sample #2	29.5	0.40	0	0.10
Sample #4	24.8	1.00	0	0.00
Average	25.8	0.50	0	0.20
Standard deviation	3.36	0.46	0	0.265
p-value compared with Cas9 sample	-	2.07×10^{-4}	1.84×10^{-4}	1.93×10^{-4}

Table S3. Sequences for ssODNs and primers

Primer Name	Sequence (5' to 3')	Description
**For vector construction**		
NLS-Acr-Cdt1 long Fw	ccgggggatccactagttctagagcGCCACCATGGACAAGAAGTAC	for construction of pEB.AcrIIA4/AcrIIA5-Cdt1-T2A-Cas9 variants (HiFiCas9, xCas9, evoCas9, SniperCas9, LZ3 Cas9, R63A/Q768A Cas9, and SpartaCas9)
pEBCas9 Fw	ccgggggatccactagttctagagcGCCACCATGGACAAGAAGTAC	for construction of pEB.HiFiCas9 and pEB.xCas9
pEBCas9 Rv	atggctgattatgatctagagtcgcTCACACCTTCCTCTTCTTCTTGG	for construction of pEB.HiFiCas9, xCas9, evoCas9, SniperCas9, LZ3 Cas9, R63A/Q768A Cas9, and SpartaCas9, pEB.AcrIIA4/AcrIIA5-Cdt1-T2A- (HiFiCas9, xCas9, evoCas9, SniperCas9, LZ3 Cas9, R63A/Q768A Cas9, and SpartaCas9)
HiFi mut R691A Fw	AAGTCCGATGGATTTGCCAACgccAACTTCATGCAGTTGATCCA	for construction pEB.HiFiCas9
HiFi mut R691A Rv	TGGATCAACTGCATGAAGTTggcGTTGGCAAATCCATCGGACTT	for HiFi Cas9 mutant plasmid
NLS-AcrCdt1-HypaHF long Fw	ccaggacaccatcgagggcagaggaagtctgctaacatgcggtgacgtcgaggaga atcctggccca gataaaaagtattctattggttta	for construction of pEB.AcrIIA4-Cdt1-T2A-HF1Cas9, pEB.AcrIIA5-Cdt1-T2A-HF1Cas9, pEB.AcrIIA4-Cdt1-T2A-HypaCas9, and pEB.AcrIIA5-Cdt1-T2A-HypaCas9
pEBHypaHFCas9 Fw	ccgggggatccactagttctagagc GCCACCATG gataaaaagtattctattggttta	for construction of pEB.HF1Cas9 and pEB.HypaCas9
pEBHypaHFCas9 Rv	atggctgattatgatctagagtcgcTCAgactttcctcttcttcttggg	for construction of pEB.HF1Cas9, pEB.AcrIIA4-T2A-HF1Cas9, pEB.AcrIIA5-T2A-HF1Cas9, pEB.AcrIIA4-Cdt1-T2A-HF1Cas9, pEB.AcrIIA5-Cdt1-T2A-HF1Cas9, pEB.HypaCas9, pEB.AcrIIA4-T2A-HypaCas9, pEB.AcrIIA5--T2A-HypaCas9, pEB.AcrIIA4-Cdt1-T2A-HypaCas9, and pEB.AcrIIA5-Cdt1-T2A-HypaCas9
NLS-AcrCdt1-eSp long Fw	ccaggacaccatcgagggcagaggaagtctgctaacatgcggtgacgtcgaggaga atcctggccca gacaagaagtacagcatcgg	for construction of pEB.AcrIIA4-Cdt1-T2A-eCas9 and pEB.AcrIIA5-Cdt1-T2A-eCas9
pEBeSpCas9 Fw	ccgggggatccactagttctagagc GCCACCATG gacaagaagtacagcatcgg	for construction of pEB.eCas9
pEBeSpCas9 Rv	atggctgattatgatctagagtcgcTCA gactttcctcttcttcttggg gtcgcctcccagctgagac	for construction of pEB.eCas9, pEB.AcrIIA4-T2A-eCas9, pEB.AcrIIA5-T2A-eCas9, pEB.AcrIIA4-Cdt1-T2A-eCas9, and pEB.AcrIIA5-Cdt1-T2A-eCas9
GA pEB NLS Fw	GGATCCACTAGTTCTAGAGCGCCACCATGGGTGGCCCG	for construction of AcrIIA4, AcrIIA5, AcrIIA4-Cdt1,and AcrIIA5-Cdt1 fragments
T2A Rv	TGGGCCAGGATTCTCCTCGA	for construction of AcrIIA4-Cdt1and AcrIIA5-Cdt1 fragments
FLAG Rv for Cdt1 remove	GACTTCCTCTGCCCTCCTTATCGTCATCGTCCTTGTAGTC	for construction of AcrIIA4 and AcrIIA5 fragments
5 end T2A Fw	GAGGGCAGAGGAAGTCTGCT	for construction of pEB-AcrIIA4/5-T2A-Cas9mutants
M495V/Y515N/K526E Fw for evoCas9	CATCGAAAGGgtgACTAACTTTGATAAAAATCTGCCTAACGAAAA GGTGCTTCCTAAACACTCTCTGCTG	for construction of pEB.evoCas9
M495V Rv for evoCas9	CAAAGTTAGTCAcCCTTTCGATGAAGGACTGGG	for construction of pEB.evoCas9
M495V/Y515N/K526E Rv for evoCas9	TCTGTGACGTATTTGACctcGGTGAGCTCGTTATAAACTGTGAAG TACTCgttCAGCAGAGAGTGTTTAGGAA	for construction of pEB.evoCas9
K526E Fw for evoCas9	TAACGAGCTCACCgagGTCAAATACGTCACAGA	for construction of pEB.evoCas9
R661L Rv for evoCas9	TTCTTGACAGaagCCCCCATCCTGTATATCGGC	for construction of pEB.evoCas9
R661L Fw for evoCas9	AGGATGGGGGcttCTGTCAAGAAAACTGATCAA	for construction of pEB.evoCas9
F539S Rv for Sniper-Cas9	CTCCAGACAGggaTGCTGGCTTTCTCATCCCTT	for construction of pEB.Sniper-Cas9
F539S Fw for Sniper-Cas9	AAAGCCAGCAtccCTGTCTGGAGAGCAGAAGAA	for construction of pEB.Sniper-Cas9
M763I Rv for Sniper-Cas9	TCTCTCGGGCtatCTCGATAACGATATTCTCGG	for construction of pEB.Sniper-Cas9
M763I Fw for Sniper-Cas9	CGTTATCGAGataGCCCGAGAGAACCAAACTAC	for construction of pEB.Sniper-Cas9
K890N Rv for Sniper-Cas9	GTGTGATCAGattGGCGTTCAGCAGCTGCCGCC	for construction of pEB.Sniper-Cas9
K890N Fw for Sniper-Cas9	GCTGAACGCCaatCTGATCACACAACGGAAGTT	for construction of pEB.Sniper-Cas9
N690C Rv for LZ3 Cas9	GAAGTTCCGgcaGGCAAATCCATCGGACTTAAG	for construction of pEB.LZ3 Cas9
N690C Fw for LZ3 Cas9	TGGATTTGCCtgcCGGAACTTCATGCAGTTGAT	for construction of pEB.LZ3 Cas9
T769I Rv for LZ3 Cas9	CCTTCTGGGTtatTTGGTTCTCTCGGGCCATCT	for construction of pEB.LZ3 Cas9
T769I Fw for LZ3 Cas9	AGAGAACCAAataACCCAGAAGGGACAGAAGAA	for construction of pEB.LZ3 Cas9
G915M Rv for LZ3 Cas9	TTTTGATGAAcatGGCTTTATCCAACTCAGACA	for construction of pEB.LZ3 Cas9
G915M Fw for LZ3 Cas9	GGATAAAGCCatgTTCATCAAAAGGCAGCTTGT	for construction of pEB.LZ3 Cas9
N980K Rv for LZ3 Cas9	CATGGTGGTActtGTTGATCTCTCTCACCTTAT	for construction of pEB.LZ3 Cas9
N980K Fw for LZ3 Cas9	AGAGATCAACaagTACCACCATGCGCATGATGC	for construction of pEB.LZ3 Cas9
R63A Rv for R63A/Q768A Cas9	TTCTTTTGAGcgcCGTGGCTTCGGCCGTCTCCC	for construction of pEB.R63A/Q768A Cas9
R63A Fw for R63A/Q768A Cas9	CGAAGCCACGgcgCTCAAAAGAACAGCACGGCG	for construction of pEB.R63A/Q768A Cas9
Q768A Rv for R63A/Q768A	TCTGGGTAGTtgcGTTCTCTCGGGCCATCTCGA	for construction of pEB.R63A/Q768A Cas9
Q768A Fw for R63A/Q768A	CCGAGAGAACgcaACTACCCAGAAGGGACAGAA	for construction of pEB.R63A/Q768A Cas9
D23A Rv for SpartaCas9	CCTTGTACTCtgcCGTAATGACGGCCCAGCCGA	for construction of pEB.SpartaCas9
D23A Fw for SpartaCas9	CGTCATTACGgcaGAGTACAAGGTGCCGAGCAA	for construction of pEB.SpartaCas9
T67L Rv for SpartaCas9	TGCGCCGTGCcagTCTTTTGAGCCGCGTGGCTT	for construction of pEB.SpartaCas9
T67L Fw for SpartaCas9	GCTCAAAAGActgGCACGGCGCAGATATACCCG	for construction of pEB.SpartaCas9
Y128V Rv for SpartaCas9	ACTTTTCATGgacCGCCACCTCGTCCACGATAT	for construction of pEB.SpartaCas9
Y128V Fw for SpartaCas9	CGAGGTGGCGgtcCATGAAAAGTACCCAACCAT	for construction of pEB.SpartaCas9
D1251G Rv for SpartaCas9	TCTGCTCATTaccTTCGGGAGACCCTTTGAGCT	for construction of pEB.SpartaCas9
D1251G Fw for SpartaCas9	GTCTCCCGAAggtAATGAGCAGAAGCAGCTGTT	for construction of pEB.SpartaCas9
**For editing assesment**		
AAVS1 ssODN	TATATTCCCAGGGCCGGTTAATGTGGCTCTGGTTCTGGGTACTTT TATCTGTCCCCTCCGGATCCAAGCTTCCACAGTGGGGCCACTAGG GACAGGATTGGTGACAGAAAAGCCCCATCCTTAGGCCTCC	for HDR template
AAVS1 target Fw	CCTCTCTGGCTCCATCGTAAGCAAA	for AAVS1 target site amplification
AAVS1 target Rv	CCGAGAGCTCAGCTAGTCTTCTTCC	for AAVS1 target site amplification
AAVS1 off-target Fw	GGGTGGGGATGCATTAGGGCTTGGT	for AAVS1 off-target site amplification
AAVS1 off-target Rv	CCCCACCCAGACATAGAACCCTTTGC	for AAVS1 off-target site amplification
EMX1 ssODN	TCCTCCCCATTGGCCTGCTTCGTGGCAATGCGCCACCGGTTGATG TGATGGGAGCCCTTGGATCCAAGCTTCTTCTGCTCGGACTCAGG CCCTTCCTCCTCCAGCTTCTGCCGTTTGTACTTTGTCCTC	for HDR template
EMX1 target Fw	GCCCTGCCATCCCCTTCTGTGAATG	for EMX1 target site amplification
EMX1 target Rv	CCAGGCCTCAGCCAGCCCATTGCTT	for EMX1 target site amplification
EMX1 off-target 1 Fw	GCCCCTCTAATACAATGGGAAGGACAGCTT	for EMX1 Off-target site 1 amplification
EMX1 off-target 1 Rv	GGGCCTGCAGACAGGAATAGCCCTA	for EMX1 Off-target site 1 amplification
EMX1 off-target 2 Fw	GGCTGCCATGGAAATTCCAGAGGGA	for EMX1 Off-target site 2 amplification
EMX1 off-target 2 Rv	GCCGGGGGTCACTGGATGAATCCTC	for EMX1 Off-target site 2 amplification
VEGFA ssODN	TATTGGAATCCTGGAGTGACCCCTGGCCTTCTCCCCGCTCCAACG CCCTCAACCCCACAGGATCCAAGCTTACACACTCACTCACCCACA CAGACACACACGTCCTCACTCTCGAAGACGCTGCTCGCT	for HDR template

VEGFA target Fw	GCCGCTCACTTTGATGTCTG	for VEGFA target site amplification
VEGFA target Rv	GAGCCGTTCCCTCTTTGCTA	for VEGFA target site amplification
VEGFA off-target 1 Fw	AACTACAAGTCTCGGCTGCC	for VEGFA Off-target site 1 amplification
VEGFA off-target 1 Rv	CCCAACCTCCAGTCCCAGG	for VEGFA Off-target site 1 amplification
VEGFA off-target 2 Fw	AGCGATTCTCCTGCCTCAACC	for VEGFA Off-target site 2 amplification
VEGFA off-target 2 Rv	CAGTATGAACGGGAGTGGTAAGGA	for VEGFA Off-target site 2 amplification
