# Supplementary material for: SpCas9-HF1 enhances accuracy of cell cycle-dependent genome editing by increasing HDR efficiency, and by reducing off-target effects and indel rates
Source: Mol Ther Nucleic Acids. 2024 Jan 23;35(1):102124. doi: 10.1016/j.omtn.2024.102124 (PMC10848011; doi:10.1016/j.omtn.2024.102124)
Supplement: Document S2. Article plus supplemental information [file mmc2.pdf]

Molecular Therapy
Nucleic Acids
Original Article

SpCas9-HF1 enhances accuracy of cell cycle-dependent genome editing by increasing HDR efficiency, and by reducing off-target effects and indel rates

Daisuke Matsumoto,[1,2] Erina Matsugi,[1] Kanae Kishi,[1] Yuto Inoue,[2] Kiyomi Nigorikawa,[1,2] and Wataru Nomura[1,2]

[1]Graduate School of Biomedical and Health Sciences, Hiroshima University, 1-2-3 Kasumi, Minami-ku, Hiroshima 734-8553, Japan; [2]School of Pharmaceutical Sciences, Hiroshima University, 1-2-3 Kasumi, Minami-ku, Hiroshima 734-8553, Japan

In genome editing, it is important to avoid off-target mutations so as to reduce unexpected side effects, especially for therapeutic applications. Recently, several high-fidelity versions of SpCas9 have been developed to reduce off-target mutations. In addition to reducing off-target effects, highly efficient intended target gene correction is also essential to rescue protein functions that have been disrupted by single nucleotide polymorphisms. Homology-directed repair (HDR) corrects genes precisely using a DNA template. Our recent development of cell cycle-dependent genome editing has shown that regulation of Cas9 activation with an anti-CRISPR-Cdt1 fusion protein increases HDR efficiency and reduces off-target effects. In this study, to apply high-fidelity SpCas9 variants to cell cycle-dependent genome editing, we evaluated anti-CRISPR inhibition of high-fidelity SpCas9s. In addition, HDR efficiency of high-fidelity SpCas9s was addressed, identifying eSpCas9, SpCas9-HF1, and LZ3 Cas9 as promising candidates. Although eSpCas9 and LZ3 Cas9 showed decreased HDR efficiency in cell cycle-dependent genome editing, SpCas9-HF1 successfully achieved increased HDR efficiency and few off-target effects when co-expressed with an AcrIIA4-Cdt1 fusion.

INTRODUCTION

Modification of target genes in various organisms is useful not only to examine gene functions, but also to endow cells with selected functions. CRISPR-Cas9 is the most widely used tool for gene modification.[1–4] It is important to edit target gene sequences precisely, without introducing mutations at off-target sites. Precise editing is achieved by increasing the editing ratio using homology-directed repair[5] (HDR). HDR is a DNA repair pathway mediated by gene-repair proteins such as CtIP and the MRN complex.[6] A customized repair template such as a donor plasmid or a single-stranded donor oligonucleotide (ssODN) is required for HDR to change nucleotide sequences. HDR acts during the S/G2 phases in the cell cycle.[7,8] Previously, our group showed that cell cycle-dependent regulation of SpCas9 with Cdt1-fused anti-CRISPR increases HDR efficiency.[9] In this system, an anti-CRISPR called AcrIIA4, derived from *Listeria monocytogenes* prophage[10] is fused with amino acid residues 30 to 120 of Cdt1 (Cdt1[30–120]) to achieve cell cycle-specific inhibition of SpCas9 activity at G1 phase. In S/G2 phases, AcrIIA4-Cdt1 is degraded via ubiquitination of its Cdt1 domain, and SpCas9 is activated.

Recently, we applied AcrIIA5 from *Streptococcus thermophilus* phage D4276[11] to cell cycle-dependent genome editing. AcrIIA5-Cdt1 increased HDR efficiency and decreased off-target effects, depending on the target DNA sequence.[12] Moreover, Cdt1-fused anti-CRISPRs have shown synergistic increases of HDR efficiency when used with SpCas9 fused with Geminin 1-110 fragment (SpCas9-Gem), which is degraded in G1 phases.[12–14] Cell cycle-dependent genome editing not only increases HDR efficiency, but also suppresses off-target effects.

We have shown that cell cycle-dependent genome editing using Cdt1-fused anti-CRISPRs is effective when combined with modified SpCas9, suggesting that it could be possible to use high-fidelity SpCas9 variants to increase precision of genome editing. Several mutants such as eSpCas9, SpCas9-HF1, HypaCas9, and R63A/Q768A Cas9 have been rationally engineered from parental SpCas9 to reduce the binding affinity of the Cas9-single guide RNA (sgRNA) complex to DNA, suppressing off-target activity.[15–18] By site-directed mutagenesis, changing amino acid residues that interact with DNA into alanine, binding affinity of these mutants to DNA is reduced. As a result, stability of SpCas9 variants and the sgRNA complex diminishes when they bind to off-target DNA sequences, and off-target effects are thereby suppressed, although target gene editing efficiency is almost equal to that of parental SpCas9. Another approach for obtaining highly specific Cas9 variants is based on directed evolution. xCas9 is another high-fidelity variant of SpCas9, which was developed using phage-assisted continuous evolution.[19] Among evolved xCas9

Received 12 April 2023; accepted 18 January 2024;
https://doi.org/10.1016/j.omtn.2024.102124.

Correspondence: Wataru Nomura, Graduate School of Biomedical and Health Sciences, Hiroshima University, 1-2-3 Kasumi Minami-ku, Hiroshima 734-8553, Japan.
E-mail: wnomura@hiroshima-u.ac.jp

This is an open access article under the CC BY license (http://creativecommons.org/licenses/by/4.0/).

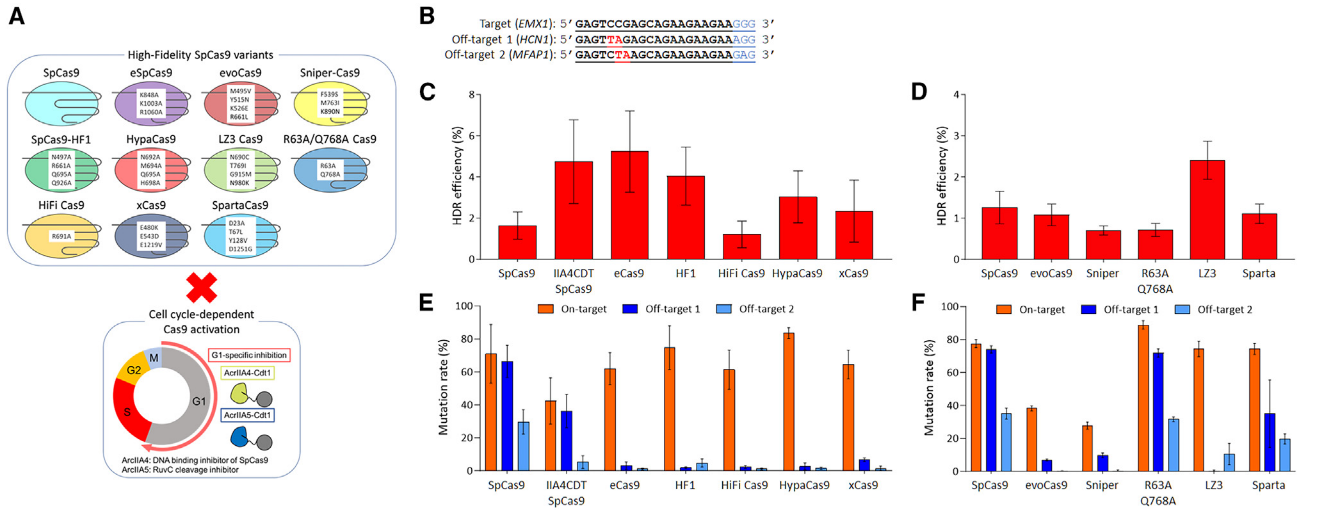

Figure 1. Comparison of HDR efficiency and on- and off-target mutation rates among SpCas9, AcrIIA4-Cdt1-T2A-SpCas9, and SpCas9 variants at the EMX1 site

(A) Schematic representation of this study. Synergistic, precise genome editing by combining high-fidelity SpCas9 variants and anti-CRISPR mediated activity control. (B) On- and off-target sequences used in experiments. (C and D) Evaluations of HDR efficiency in the first (C) and the second (D) screenings. (E and F) Evaluations of on- and off-target mutation rates in the first (E) and the second (F) screenings. HDR efficiency was evaluated using HindIII digestion for PCR products amplified from target sites in extracted genomic DNA. HDR efficiency was calculated using the following formula: $100 \times ((b + c)/(a + b + c))$, where "a" is the integrated intensity of undigested PCR product, and "b" and "c" are integrated intensities of each cleavage product. On- and off-target mutation rates were analyzed by TIDE, utilizing Sanger sequencing data of PCR products amplified from target sites in extracted genomic DNA. On-target mutations include mutagenesis by a-EJ and NHEJ. Samples are identified by names under graph bars. IIA4CDTeSpCas9 means co-expression of AcrIIA4-Cdt1 and eSpCas9. n = 4 except for xCas9(3.7) off-target 1 mutation sample. n = 3 for xCas9(3.7) off-target 1 mutation sample. Significance of differences was tested with Student's t test by comparing each sample independently with SpCas9. p values are in Table S1.

variants, xCas9(3.7) showed the best target specificity with broad PAM recognition and fewer unintended mutations. SpartaCas9 was also evolved with phage selection by introducing mutations using scanning mutagenesis of oligo-directed targets (SMOOT).[20] An evoCas9 was evolved from SpCas9 by adding random mutations in the REC3 domain and screening in yeast.[21] HiFi Cas9 and SniperCas9 were evolved in *E. coli* by introducing target gene sequences in the human genome into the *E. coli* genome and screening with plasmid DNA, which encodes an antibiotic resistance gene and an off-target DNA sequence.[22,23] An LZ3 Cas9 is screened in HEK293FT cells with tagmentation-based tag integration site sequencing.[24] In this study, we checked HDR activity of 10 high-fidelity Cas9 variants, eSpCas9, SpCas9-HF1, HiFi Cas9, HypaCas9, xCas9(3.7), evoCas9, Sniper-Cas9, LZ3 Cas9, R63A/Q768A Cas9, and SpartaCas9 at the EMX1 site and selected optimal Cas9 variants to apply to cell cycle-dependent genome editing to improve precision.

RESULTS

Inhibition of SpCas9 variant activity by AcrIIA4 and AcrIIA5

First, inhibition activity of AcrIIA4 and AcrIIA5 against engineered or evolved SpCas9 variants, eSpCas9, SpCas9-HF1, HiFi Cas9, HypaCas9, and xCas9(3.7), was compared with that of parental SpCas9 (Figure S1A). As the second screening, inhibition activity of AcrIIA4 and AcrIIA5 against evoCas9, Sniper-Cas9, LZ3 Cas9, R63A/Q768A Cas9, and SpartaCas9 was also assessed (Figure S1B). Episomal expression vectors encoding genes of anti-CRISPR and SpCas9 variants sectioned by the T2A self-cleaving peptide sequence were constructed to obtain equal amounts of anti-CRISPR and SpCas9 variants in cells. Genome editing efficiency was evaluated as follows. Briefly, after transfection with vectors, 293A cells were selected with hygromycin for 3 days. Then cells were transfected with an sgRNA coding vector targeting the *EMX1* gene by lipofection. Genomic DNA was extracted 48 h after the second transfection. Target mutation efficiency was calculated using the Tracking of Indels by Decomposition (TIDE) program (https://tide.nki.nl/).[25] Target mutation efficiency of each SpCas9 variant was almost the same as that of parental SpCas9 and both AcrIIA4 and AcrIIA5 efficiently inhibited SpCas9 variants.

Comparison of HDR and on/off-target efficiencies of SpCas9 variants in the presence of ssODN

As there is relatively little information about HDR efficiency of high-fidelity SpCas9 variants, genome editing efficiency, including on/off-target mutations, was evaluated using ssODN as the donor DNA. In these experiments, selection with hygromycin after the first transfection was continued for 4–5 days, and the sgRNA encoding vector and ssODNs having an HindIII site were introduced into selected cells by electroporation. HDR efficiency was assessed by HindIII digestion of PCR-amplified fragments from the EMX-1 target site (Figure 1B). Interestingly, eSpCas9 and SpCas9-HF1 showed more than 2-fold higher HDR activity than parental SpCas9 (Figure 1C). LZ3 Cas9 also showed higher HDR activity compared with SpCas9 in the second screening (Figure 1D). HDR efficiency was almost the same as

that of cell cycle-dependent activation of SpCas9 using AcrIIA4-Cdt1 in eCas9 and SpCas9-HF1. Other SpCas9 variants, HiFi Cas9, HypaCas9, xCas9(3.7), evoCas9, Sniper-Cas9, R63A/Q768A Cas9, and SpartaCas9 showed similar or lower HDR efficiency than SpCas9. There was no difference in on-target mutation efficiency among parental SpCas9 and high-fidelity SpCas9 variants (Figure 1E). All SpCas9 variants reduced off-target mutations to less than 20% compared with parental SpCas9, as previously reported (Figure 1E). Cell cycle-dependent activation of SpCas9 using AcrIIA4-Cdt1 also reduces off-target effects, but high-fidelity SpCas9 variants (except for R63A/Q768A Cas9 and SpartaCas9) reduced off-target effects more strongly (Figures 1E and 1F). Mutation positions of eSpCas9 are K848A, K1003A, and R1060A at RuvC and HNH nuclease domains.[15] For SpCas9-HF1, mutations, N497A, R661A, Q695A, and Q926A, are introduced into the REC1 and RuvC nuclease domains.[14] LZ3 Cas9 has an N690C mutation in REC III and T769I, G915M, and N980K mutations in the RuvC nuclease domain. These mutations reduce interactions between SpCas9 variants and target DNA strands because tertiary complexes of SpCas9 variants, sgRNA, and DNA are destabilized. In eSpCas9, it may be that a weakened interaction between eSpCas9 and the non-target DNA strand affects the location of the cleavage site by RuvC. Although the reason that HDR efficiency is increased by eSpCas9, SpCas9-HF1, and LZ3 Cas9 remains unclear, these results support the hypothesis that a combination of cell cycle-dependent activation and eSpCas9, SpCas9-HF1, or LZ3 Cas9 may synergistically increase HDR and reduce off-target effects.

Increasing the HDR ratio by combining AcrIIA4-Cdt1 with SpCas9-HF1

As the next step, effects of co-expression of AcrIIA4-Cdt1 and AcrIIA5-Cdt1 with eSpCas9, SpCas9-HF1, or LZ3 Cas9 on HDR efficiency and off-target effects were addressed. Episomal expression vectors were constructed as described above. HindIII digestion for evaluation of HDR efficiency and Sanger sequencing and TIDE analysis for evaluation of on-target mutations were performed utilizing three target sites, AAVS1, EMX1, and VEGFA. At the AAVS1 target site, increased HDR efficiency was not observed for eSpCas9, SpCas9-HF1, and LZ3 Cas9 (Figures 2A and 2D). In addition, when eSpCas9 was used with anti-CRISPR+Cdt1, HDR efficiency was decreased, especially with AcrIIA5+Cdt1, compared with SpCas9. The eSpCas9 on-target mutation rate was decreased in combination with AcrIIA4-Cdt1 or AcrIIA5-Cdt1 as well. SpCas9-HF1 showed increased HDR efficiency with AcrIIA4-Cdt1 (1.86-fold) or AcrIIA5-Cdt1 (1.74-fold) compared with SpCas9-HF1 alone, although it was almost the same as that of SpCas9. Decreased off-target effects were observed for all samples, eSpCas9 or SpCas9-HF1 alone, and those combined with AcrIIA4-Cdt1 and AcrIIA5-Cdt1, compared with SpCas9. The accuracy of targeted gene correction, calculated by dividing HDR efficiency by the on-target mutation rate, increased up to 3.77-fold for combinations of AcrIIA4-Cdt1 and SpCas9-HF1, compared with SpCas9 (Table 1).

At the EMX1 site, increased HDR efficiency was observed for eSpCas9, SpCas9-HF1, and LZ3 Cas9 alone, as observed in other experiments (Figures 2B and 2E). However, eSpCas9 did not improve HDR efficiency when combined with AcrIIA4-Cdt1 or AcrIIA5-Cdt1. For SpCas9-HF1, it showed a 1.55-fold increase of HDR efficiency when used with AcrIIA4-Cdt1, compared with SpCas9-HF1 alone, although this improvement was not observed when used with AcrIIA5-Cdt1. In the case of LZ3 Cas9, AcrIIA4-Cdt1 and AcrIIA5-Cdt1 did not improve the HDR ratio as eSpCas9 did. Numerous off-target effects were observed at the EMX1 site with SpCas9, but a significant decrease was shown for all samples with eSpCas9 or SpCas9-HF1 alone and those combined with AcrIIA4-Cdt1 or AcrIIA5-Cdt1. In addition, the on-target mutation rate was suppressed when eSpCas9 or SpCas9-HF1 was used in combination with AcrIIA4-Cdt1 or AcrIIA5-Cdt1.

Finally, at the VEGFA site, eSpCas9 or SpCas9-HF1 alone did not improve HDR efficiency, but when combined with AcrIIA4-Cdt1, they improved it by 1.3-fold and 2.2-fold, respectively (Figure 2C). On the other hand, LZ3 Cas9 alone showed improvement of HDR efficiency at the VEGFA site, but enhancement of HDR efficiency was not observed when combining AcrIIA4-Cdt1 (Figure 2F). In the AcrIIA5-Cdt1 combination, eSpCas9 and LZ3 Cas9 significantly reduced HDR efficiency, while SpCas9-HF1 increased it 1.4-fold. Reduction of on-target mutation rates was observed for eSpCas9, SpCas9-HF1, or LZ3 Cas9 when they were combined with AcrIIA4-Cdt1 or AcrIIA5-Cdt1 as other sites. For off-target effects, eSpCas9 alone showed a 50% mutation rate for off-target 1, but it was significantly reduced when combined with AcrIIA4-Cdt1. This tendency was also seen with LZ3 Cas9, and for off-target 1. Off-target effects were further reduced when eSpCas9 and SpCas9-HF1 were combined with AcrIIA5-Cdt1. Results were evaluated by HDR efficiency, on-target mutation rate, and off-target mutation rate for three target sites. Sequence dependence was observed in part, but the important finding is that on-target mutation rates were reduced by a combination of high-fidelity SpCas9s and anti-CRISPR+Cdt1, which was not observed when SpCas9 was combined with anti-CRISPR+Cdt1.[9] The reduction of on-target mutation may indicate that these high-fidelity SpCas9s are effectively suppressed by anti-CRISPR+Cdt1 during G1 phase. A combination of SpCas9-HF1 and AcrIIA4-Cdt1 increases HDR efficiency, but eSpCas9 and LZ3 Cas9 does not.

DISCUSSION

SpCas9 cleaves the target DNA sequence three-base from the PAM and the DNA end is blunt-ended[26] or staggered by 1 base pair (bp).[1,27] Ku70, which is a component of the Ku70-Ku80 heterodimer that works as a non-homologous DNA end joining (NHEJ) mediator, binds more poorly to single-stranded DNA (ssDNA) overhangs than to blunt ends in yeast.[28] The preferential binding of Ku protein to double-stranded DNA (dsDNA) may lead to NHEJ-mediated repair by SpCas9 after cleavage. Although eSpCas9, SpCas9-HF1, and LZ3 Cas9 showed increased HDR efficiency on their own, they should not be able to escape the NHEJ repair pathway by making a long overhang dsDNA at the cleavage site, like the 30-bp ssDNA 3′ overhung dsDNA used in Ku-DNA binding experiments. Thus, involvement of other factors in the mechanism should be considered. As shown in our recent report using AcrIIA5-Cdt1 with SpCas9,[12] we also observed that AcrIIA4-Cdt1 with SpCas9-HF1 increased HDR

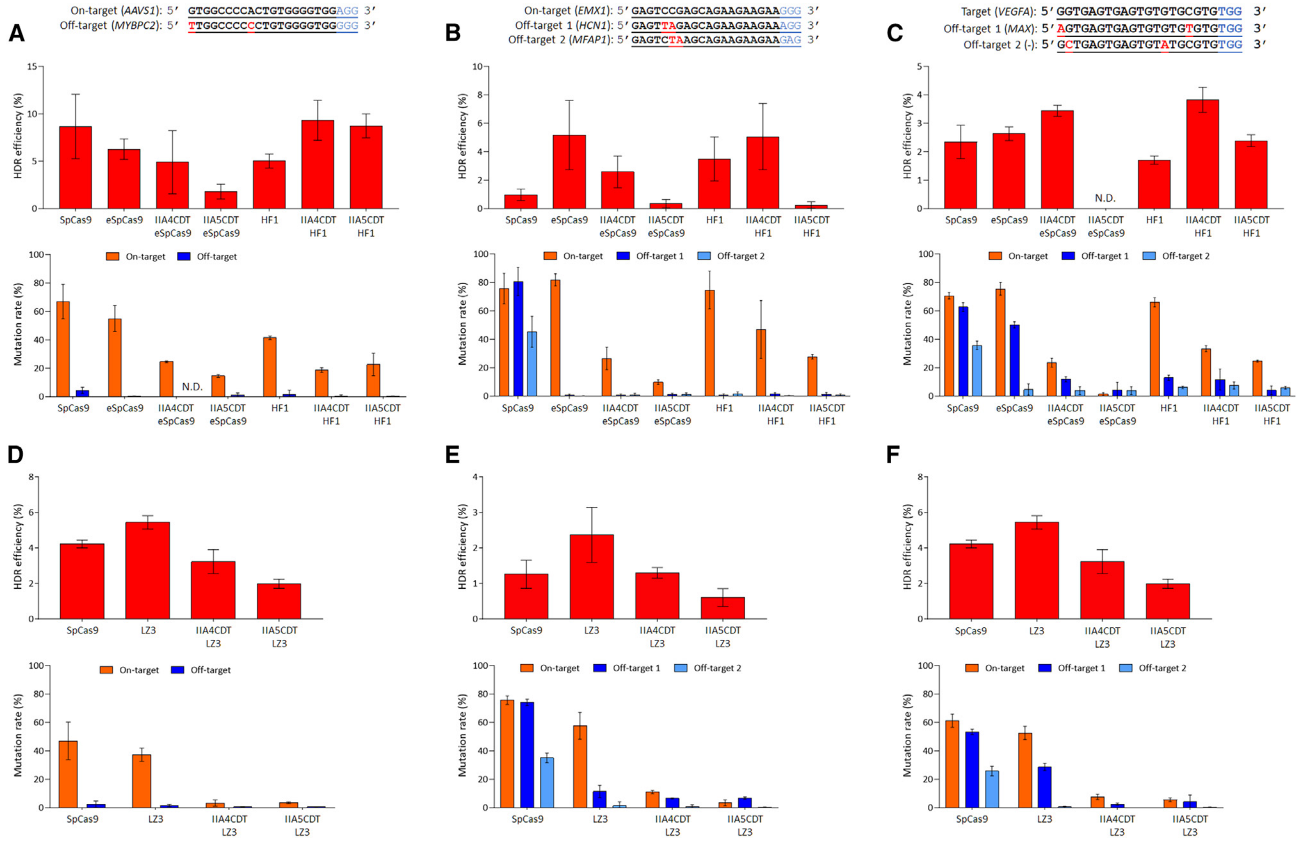

Figure 2. Comparison of HDR efficiency and on- and off-target mutation rates among SpCas9, eSpCas9, AcrIIA4-Cdt1-T2A-eSpCas9, AcrIIA5-Cdt1-T2A-eSpCas9, SpCas9-HF1, AcrIIA4-Cdt1-T2A-SpCas9-HF1, AcrIIA5-Cdt1-T2A-SpCas9-HF1, LZ3 Cas9, AcrIIA4-Cdt1-T2A-LZ3 Cas9, and AcrIIA5-Cdt1-T2A LZ3 Cas9 at the AAVS1

(A and D), EMX1 (B and E), and VEGFA (C and F) sites. Samples are identified by names under graph bars. IIA4CDT or IIA5CDT and SpCas9 variant names mean co-expression of AcrIIA4-Cdt1 or AcrIIA5-Cdt1 with indicated SpCas9 variants. HDR efficiency was evaluated using HindIII digestion for PCR products amplified from target sites in extracted genomic DNA. HDR efficiency was calculated with the following formula: $100 \times ((b + c)/(a + b + c))$, where "a" is the integrated intensity of undigested PCR product, and "b" and "c" are integrated intensities of each cleavage product. On- and off-target mutation rates were analyzed with TIDE utilizing Sanger sequencing data of PCR products amplified from target sites in extracted genomic DNA. On-target mutations include mutagenesis by a-EJ and NHEJ. n = 4 for AAVS1 and EMX1 target samples. n = 3 for VEGFA target samples. N.D., not determined. Significance in difference was tested by Student's t test by comparing each sample independently with SpCas9. p values are in Table S2.

efficiency at all target sites and that AcrIIA5-Cdt1 with SpCas9-HF1 failed at the EMX1 site. Success of SpCas9 activity control by AcrIIA5-Cdt1 may depend on target sequences, especially in relation to their melting temperatures (Tm). Calculation of GC content (%) and Tm values (°C) of target sites with SnapGene software found 75% GC and 66°C for the AAVS1 site, 50% GC and 55°C for the EMX1 site, and 60% GC and 60°C for the VEGFA site. These results suggest that AcrIIA5-Cdt1 shows cell cycle-dependent control of SpCas9 or its variants when the Tm of a target sequence is high (about 65°C) and results in increased HDR efficiency. In contrast, when the Tm of a target sequence is relatively low, AcrIIA5-Cdt1 only inhibits SpCas9 or its variants. This may be because AcrIIA5 inhibits SpCas9 by binding to the ternary complex of target DNA-Cas9-sgRNA, and not to the Cas9-sgRNA complex like AcrIIA4. When the Tm of the target sequence is high, the SpCas9-sgRNA-DNA complex may be stabilized, cleaving the target DNA efficiently. Above all, AcrIIA4-Cdt1, as an excellent modulator of SpCas9 or SpCas9-HF1, can increase HDR efficiency without target sequence bias, which may result from inhibiting Cas9 DNA binding, but AcrIIA5-Cdt1 is superior to AcrIIA4-Cdt1 in that it has fewer off-target effects.

eSpCas9, SpCas9-HF1, and LZ3 Cas9 increased HDR efficiency on their own, but only SpCas9-HF1 increased HDR efficiency when combined with an anti-CRISPR+Cdt1 fusion. This may be because the mutation points in eSpCas9 and LZ3 Cas9 affect the interaction of the eSpCas9/LZ3 Cas9-sgRNA complex with anti-CRISPRs. The protein expression level of eSpCas9 and SpCas9-HF1 was not changed even when co-expressed with AcrIIA4-Cdt1 or AcrIIA5-Cdt1 in the presence or absence of sgRNA (Figure S2). This supports the conclusion that reduced HDR efficiency of eSpCas9 with anti-CRISPR+Cdt1 is not caused by eSpCas9 degradation as a result of binding ubiquitinated Cdt1. The crystal structure of SpCas9-sgRNA in complex with AcrIIA4 suggests that AcrIIA4

Table 1. Comparison of precision while using AAVS1, EMX1, and VEGFA target sgRNA among SpCas9, eSpCas9, AcrIIA4-Cdt1-T2A-eSpCas9, AcrIIA5-Cdt1-T2A-eSpCas9, SpCas9-HF1, AcrIIA4-Cdt1-T2A-SpCas9-HF1, AcrIIA5-Cdt1-T2A-SpCas9-HF1, LZ3 Cas9, AcrIIA4-Cdt1-T2A-LZ3 Cas9, and AcrIIA5-Cdt1-T2A-LZ3 Cas9

Editing precision (HDR/on-target mutation ratio)	AAVS1 target	EMX1 target	VEGFA target
SpCas9	0.13 ± 0.03	0.01 ± 0.004	0.03 ± 0.01
eSpCas9	0.12 ± 0.03	0.07 ± 0.03	0.04 ± 0.01
AcrIIA4-Cdt1-T2A-eSpCas9	0.20 ± 0.14	0.11 ± 0.03	0.15 ± 0.03
AcrIIA5-Cdt1-T2A-eSpCas9	0.12 ± 0.04	0.03 ± 0.04	N.D.
SpCas9-HF1	0.12 ± 0.02	0.05 ± 0.01	0.03 ± 0.003
AcrIIA4-Cdt1-T2A-SpCas9-HF1	0.49 ± 0.09	0.15 ± 0.08	0.12 ± 0.02
AcrIIA5-Cdt1-T2A-SpCas9-HF1	0.43 ± 0.20	0.01 ± 0.01	0.10 ± 0.01
LZ3 Cas9	0.08 ± 0.02	0.04 ± 0.01	0.10 ± 0.004
AcrIIA4-Cdt1-T2A-LZ3 Cas9	0.23 ± 0.04	0.12 ± 0.01	0.46 ± 0.22
AcrIIA5-Cdt1-T2A-LZ3 Cas9	0.30 ± 0.06	0.30 ± 0.28	0.37 ± 0.09

N.D., not determined.

interacts with the RuvC-III, Topo, and CTD domains of SpCas9.[29] In comparing mutated residues of eSpCas9 and SpCas9-HF1, the K1003A and R1060A mutations of eSpCas9 are in the RuvC-III domain, but only one Q926A mutation of SpCas9-HF1 is. LZ3 Cas9 also has G915M and N980K mutations in the RuvC-III domain. Thus, these mutations in eSpCas9 and LZ3 Cas9 may affect their interaction with AcrIIA4, resulting in different HDR efficiency between eSpCas9 and SpCas9-HF1. AcrIIA5 has been reported as a broad-spectrum inhibitor against Type II Cas9.[30] The detailed mechanism of inhibition by AcrIIA5 has not yet been identified, but it has been suggested that the N-terminal intrinsically disordered region is a critical domain for inhibition.[5] Further investigation into the structure and inhibition mechanism of AcrIIA5 is needed to explain why there is a difference in HDR efficiency, depending on target sequences, when using AcrIIA5-Cdt1 with eSpCas9, SpCas9-HF1, or LZ3 Cas9.

MATERIALS AND METHODS

Plasmid construction

pEBMulti-Hyg was purchased from FUJIFILM. The gRNA Cloning Vector and SpCas9 were gifts from George Church (Addgene plasmids # 41824 and 41815). eSpCas9(1.1) was a gift from Feng Zhang (Addgene plasmid # 71814). A BPK4410 - human expression plasmid for SpCas9 Cluster 1 (HypaCas9) was a gift from Jennifer Doudna and Keith Joung (Addgene plasmid # 101178). xCas9(3.7) was a gift from David Liu (Addgene plasmid # 108379). A HiFi Cas9 was constructed from a SpCas9 plasmid by introducing an R691A mutation with primers, HiFi mut R691A Fw, and HiFi mut R691A Rv. Plasmids for evoCas9, Sniper-Cas9, LZ3 Cas9, and R63A/Q768A Cas9 expression were constructed by introducing mutations indicated in Figure 1A to the SpCas9 plasmid. Each SpCas9 variant gene was amplified with KOD one polymerase (TOYOBO) and introduced into NotI digested pEB vector using the Gibson Assembly Master Mix (NEB). Assembled plasmids were transformed into NEB stable competent cells (NEB). For construction of AcrIIA4/5-Cdt1-T2A-SpCas9 variants, AcrIIA4-Cdt1 and AcrIIA5-Cdt1 fragments were amplified from pEB.AcrIIA4-Cdt1-T2A-SpCas9 and pEB.AcrIIA5-Cdt1-T2A-SpCas9.[8,11] AcrIIA4-Cdt1 or AcrIIA5-Cdt1 fragment was assembled with each SpCas9 variant and introduced into the pEB vector by Gibson Assembly.

Cell culture and transfection

293A cells (Invitrogen) were maintained in DMEM supplemented with 10% fetal bovine serum and penicillin/streptomycin at 37°C in an atmosphere of 5% CO_2. After introduction of the episomal vector encoding SpCas9 variants, AcrIIA4-Cdt1-T2A-SpCas9 variants, or AcrIIA5-Cdt1-T2A-SpCas9 variants using Lipofectamine 3000 (Thermo Fisher Scientific), cells were selected using 350 μg/mL Hygromycin solution (FUJIFILM) for 3 days when lipofection was used for introduction of sgRNA plasmid and for 4–5 days when electroporation was used for introduction of sgRNA plasmid and ssODN. A Neon Transfection System 10 μL Kit (Thermo Fisher Scientific) was used for electroporation. In the HDR assessment using ssODN as the template, 50 pmol of ssODN and 250 ng of sgRNA plasmid were used for 1×10^5 cells and conditions for electroporation were as follows: a pulse voltage of 1,245 V, pulse width of 10 ms, and three pulses.

Restriction enzyme assay and TIDE analysis

Forty-eight hours after transfection, genomic DNA was extracted using a QIAamp DNA Mini Kit (Qiagen). Genomic DNA (100 ng) was amplified using Tks Gflex DNA polymerase (TaKaRa) with T7E1 primers of each target. PCR conditions were 94°C for 2 min for the first denaturation, pre-amplification using 10 cycles of 98°C for 10 s, 68°C–63.5°C (−0.5°C per a cycle) for 15 s, and 68°C for 30 s, amplification using 25 cycles of 98°C for 10 s, 63°C for 15 s, and 68°C for 30 s, and a final extension at 68°C for 1 min. PCR-fragmented DNA was purified using a QIAquick PCR Purification Kit (Qiagen). Fragmented DNA (100 ng) was prepared for Sanger sequencing with a primer, and sequencing was performed by Eurofins Genomics. Sequence data were analyzed with TIDE.[25]

In the restriction enzyme assay, 200 ng of amplified DNA was reacted with 0.5 μL of HindIII (NEB) in Cutsmart buffer (NEB) at 37°C for 1 h. After the reaction, HindIII was inactivated at 65°C for 20 min. Reacted samples were analyzed using MultiNA. The indel efficiency of indel was calculated as:

$$100 * ((a + b) / (a + b + c)),$$

where a and b represent areas of cleaved fragments and c represents the area of an uncleaved fragment.

DATA AND CODE AVAILABILITY

Data that support the findings of this study are available in the supplemental information.

SUPPLEMENTAL INFORMATION

Supplemental information can be found online at https://doi.org/10.1016/j.omtn.2024.102124.

ACKNOWLEDGMENTS

We thank Sayuri Kobayashi for excellent technical assistance with experiments and Yoko Hayashi for excellent technical assistance with FACS sorting. We also thank Dr. Steven D. Aird for English language editing. This work was supported in part by the New Energy and Industrial Technique Development Organization (NEDO) of Japan, the Japan Society for the Promotion of Science (JSPS) KAKENHI (JP22H02201, JP19H02827 and JP20K21253 to W.N., JP21K14740 to D.M.), and Grants-in-Aid for Scientific Research on Innovative Areas (JP16H01420 (Resonance Bio) to W.N.), funding from Core Research for Organelle Diseases at Hiroshima University (the MEXT program to enhance Japanese research universities), HIRAKU-Global Program, which is funded by MEXT's "Strategic Professional Development Program for Young Researchers" (to D.M.), JST SPRING (JPMJSP2132 to E.M. and K.K.), funding from Takeda Science Foundation (to W.N.), the Naito Foundation (to W.N.), the Uehara Memorial Foundation (to D.M. and W.N.), the Mochida Memorial Foundation (to D.M. and W.N.), and the Suzuken Memorial Foundation (to W.N.).

AUTHOR CONTRIBUTIONS

D.M. conceived and designed the study. D.M., E.M., K.K., and Y.I. conducted experiments. D.M. performed experiments for Figures 2A–2C and S1. K.K. performed experiments for Figure S2. E.M. performed experiments for Figures 1C and 1E. Y.I. performed experiments for Figures 1D, 1F, and 2D–2F. K.N. and W.N. suggested experimental designs. W.N. supervised the project. D.M. and W.N. wrote the manuscript.

DECLARATION OF INTERESTS

The authors declare no conflicts of interest.

REFERENCES

1. Jinek, M., Chylinski, K., Fonfara, I., Hauer, M., Doudna, J.A., and Charpentier, E. (2012). A programmable dual-RNA-guided DNA endonuclease in adaptive bacterial immunity. Science *337*, 816–821.

2. Cong, L., Ran, F.A., Cox, D., Lin, S., Barretto, R., Habib, N., Hsu, P.D., Wu, X., Jiang, W., Marraffini, L.A., and Zhang, F. (2013). Multiplex genome engineering using CRISPR/Cas systems. Science *339*, 819–823.

3. Jinek, M., East, A., Cheng, A., Lin, S., Ma, E., and Doudna, J. (2013). RNA-programmed genome editing in human cells. Elife *2*, e00471.

4. Mali, P., Yang, L., Esvelt, K.M., Aach, J., Guell, M., DiCarlo, J.E., Norville, J.E., and Church, G.M. (2013). RNA-guided human genome engineering via Cas9. Science *339*, 823–826.

5. An, S.Y., Ka, D., Kim, I., Kim, E.H., Kim, N.K., Bae, E., and Suh, J.Y. (2020). Intrinsic disorder is essential for Cas9 inhibition of anti-CRISPR AcrIIA5. Nucleic Acids Res. *48*, 7584–7594.

6. Shrivastav, M., De Haro, L.P., and Nickoloff, J.A. (2008). Regulation of DNA double-strand break repair pathway choice. Cell Res. *18*, 134–147.

7. Heyer, W.D., Ehmsen, K.T., and Liu, J. (2010). Regulation of homologous recombination in eukaryotes. Annu. Rev. Genet. *44*, 113–139.

8. Hustedt, N., and Durocher, D. (2016). The control of DNA repair by the cell cycle. Nat. Cell Biol. *19*, 1–9.

9. Matsumoto, D., Tamamura, H., and Nomura, W. (2020). A cell cycle-dependent CRISPR-Cas9 activation system based on an anti-CRISPR protein shows improved genome editing accuracy. Commun. Biol. *3*, 601.

10. Rauch, B.J., Silvis, M.R., Hultquist, J.F., Waters, C.S., McGregor, M.J., Krogan, N.J., and Bondy-Denomy, J. (2017). Inhibition of CRISPR-Cas9 with Bacteriophage Proteins. Cell *168*, 150–158.e10.

11. Hynes, A.P., Rousseau, G.M., Lemay, M.L., Horvath, P., Romero, D.A., Fremaux, C., and Moineau, S. (2017). An anti-CRISPR from a virulent streptococcal phage inhibits Streptococcus pyogenes Cas9. Nat. Microbiol. *2*, 1374–1380.

12. Matsumoto, D., Kishi, K., Matsugi, E., Inoue, Y., Nigorikawa, K., and Nomura, W. (2023). Cas9-Geminin and Cdt1-fused anti-CRISPR protein synergistically increase editing accuracy. FEBS Lett. *597*, 985–994.

13. Gutschner, T., Haemmerle, M., Genovese, G., Draetta, G.F., and Chin, L. (2016). Post-translational Regulation of Cas9 during G1 Enhances Homology-Directed Repair. Cell Rep. *14*, 1555–1566.

14. Howden, S.E., McColl, B., Glaser, A., Vadolas, J., Petrou, S., Little, M.H., Elefanty, A.G., and Stanley, E.G. (2016). A Cas9 Variant for Efficient Generation of Indel-Free Knockin or Gene-Corrected Human Pluripotent Stem Cells. Stem Cell Rep. *7*, 508–517.

15. Kleinstiver, B.P., Pattanayak, V., Prew, M.S., Tsai, S.Q., Nguyen, N.T., and Joung, J.K. (2016). High-Fidelity CRISPR-Cas9 Nucleases with No Detectable Genome-Wide Off-Target Effects. Mol. Ther. *24*, S288.

16. Slaymaker, I.M., Gao, L., Zetsche, B., Scott, D.A., Yan, W.X., and Zhang, F. (2016). Rationally engineered Cas9 nucleases with improved specificity. Science *351*, 84–88.

17. Chen, J.S., Dagdas, Y.S., Kleinstiver, B.P., Welch, M.M., Sousa, A.A., Harrington, L.B., Sternberg, S.H., Joung, J.K., Yildiz, A., and Doudna, J.A. (2017). Enhanced proofreading governs CRISPR-Cas9 targeting accuracy. Nature *550*, 407–410.

18. Bratovič, M., Fonfara, I., Chylinski, K., Gálvez, E.J.C., Sullivan, T.J., Boerno, S., Timmermann, B., Boettcher, M., and Charpentier, E. (2020). Bridge helix arginines play a critical role in Cas9 sensitivity to mismatches. Nat. Chem. Biol. *16*, 587–595.

19. Hu, J.H., Miller, S.M., Geurts, M.H., Tang, W., Chen, L., Sun, N., Zeina, C.M., Gao, X., Rees, H.A., Lin, Z., and Liu, D.R. (2018). Evolved Cas9 variants with broad PAM compatibility and high DNA specificity. Nature *556*, 57–63.

20. Cerchione, D., Loveluck, K., Tillotson, E.L., Harbinski, F., DaSilva, J., Kelley, C.P., Keston-Smith, E., Fernandez, C.A., Myer, V.E., Jayaram, H., and Steinberg, B.E. (2020). SMOOT libraries and phage-induced directed evolution of Cas9 to engineer reduced off-target activity. PLoS One *15*, e0231716.

21. Casini, A., Olivieri, M., Petris, G., Montagna, C., Reginato, G., Maule, G., Lorenzin, F., Prandi, D., Romanel, A., Demichelis, F., et al. (2018). A highly specific SpCas9 variant is identified by *in vivo* screening in yeast. Nat. Biotechnol. *36*, 265–271.

22. Lee, J.K., Jeong, E., Lee, J., Jung, M., Shin, E., Kim, Y.H., Lee, K., Jung, I., Kim, D., Kim, S., and Kim, J.S. (2018). Directed evolution of CRISPR-Cas9 to increase its specificity. Nat. Commun. *9*, 3048.

23. Vakulskas, C.A., Dever, D.P., Rettig, G.R., Turk, R., Jacobi, A.M., Collingwood, M.A., Bode, N.M., McNeill, M.S., Yan, S., Camarena, J., et al. (2018). A high-fidelity Cas9 mutant delivered as a ribonucleoprotein complex enables efficient gene editing in human hematopoietic stem and progenitor cells. Nat. Med. *24*, 1216–1224.

24. Schmid-Burgk, J.L., Gao, L., Li, D., Gardner, Z., Strecker, J., Lash, B., and Zhang, F. (2020). Highly Parallel Profiling of Cas9 Variant Specificity. Mol. Cell *78*, 794–800.e8.

25. Brinkman, E.K., Chen, T., Amendola, M., and van Steensel, B. (2014). Easy quantitative assessment of genome editing by sequence trace decomposition. Nucleic Acids Res. *42*, e168.

26. Gasiunas, G., Barrangou, R., Horvath, P., and Siksnys, V. (2012). Cas9-crRNA ribonucleoprotein complex mediates specific DNA cleavage for adaptive immunity in bacteria. Proc. Natl. Acad. Sci. USA *109*, E2579–E2586.

27. Tsai, S.Q., and Joung, J.K. (2016). Defining and improving the genome-wide specificities of CRISPR-Cas9 nucleases. Nat. Rev. Genet. *17*, 300–312.

28. Foster, S.S., Balestrini, A., and Petrini, J.H.J. (2011). Functional interplay of the Mre11 nuclease and Ku in the response to replication-associated DNA damage. Mol. Cell Biol. *31*, 4379–4389.

29. Yang, H., and Patel, D.J. (2017). Inhibition Mechanism of an Anti-CRISPR Suppressor AcrIIA4 Targeting SpyCas9. Mol. Cell *67*, 117–127.e5.

30. Song, G., Zhang, F., Zhang, X., Gao, X., Zhu, X., Fan, D., and Tian, Y. (2019). AcrIIA5 Inhibits a Broad Range of Cas9 Orthologs by Preventing DNA Target Cleavage. Cell Rep. *29*, 2579–2589.e4.

OMTN, Volume *35*

Supplemental information

SpCas9-HF1 enhances accuracy of cell cycle-dependent genome editing by increasing HDR efficiency, and by reducing off-target effects and indel rates

Daisuke Matsumoto, Erina Matsugi, Kanae Kishi, Yuto Inoue, Kiyomi Nigorikawa, and Wataru Nomura

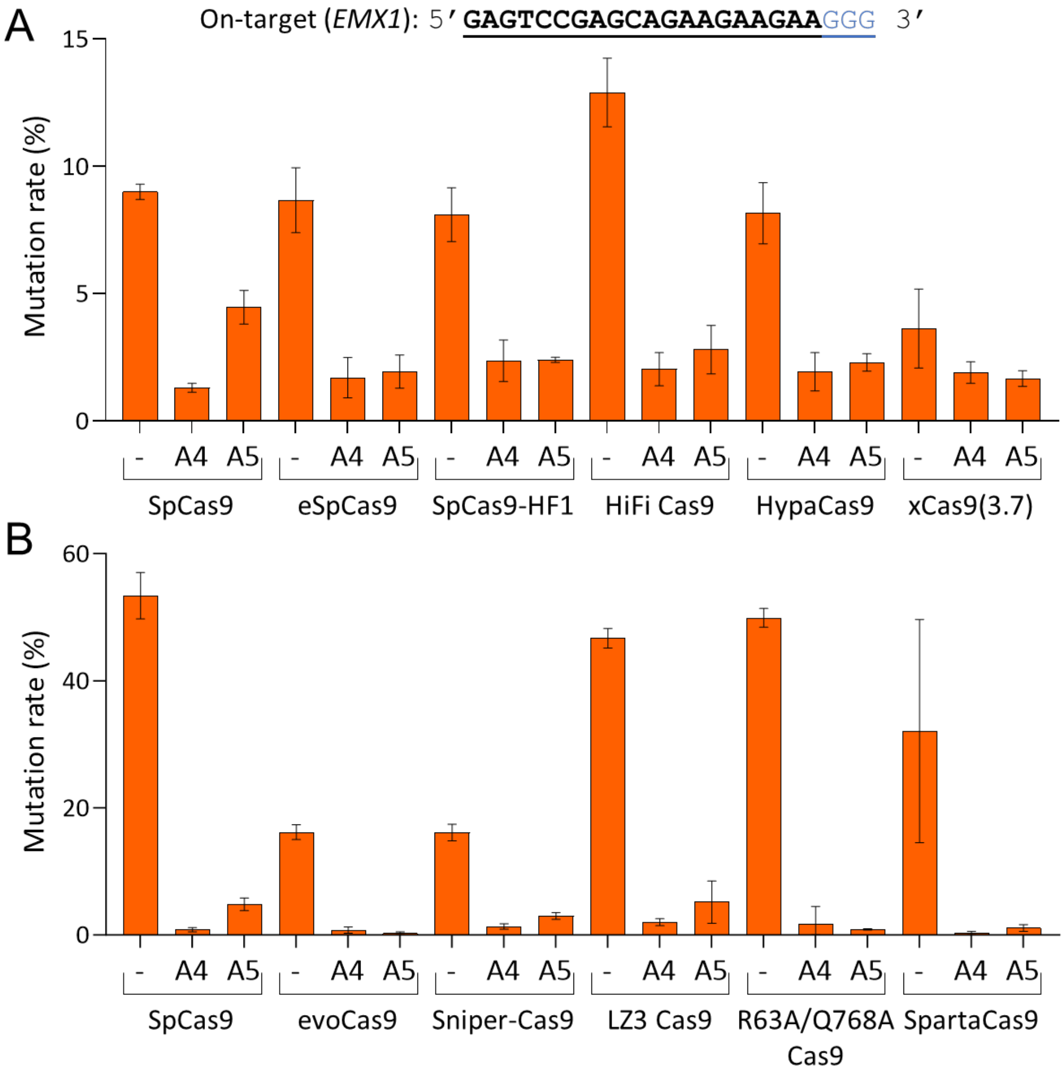

Figure S1. Evaluation of inhibition of Cas9 variants by AcrIIA4 and AcrIIA5. Mutation rates for Cas9 variants utilized in the first (A) and the second (B) screenings. The on-target DNA sequence (EMX1 site) is shown above the graph. Each PCR product amplified from extracted genomic DNA was sequenced and analyzed using TIDE. Graph bars show absence of anti-CRISPR (-) or in presence of AcrIIA4 (A4) and AcrIIA5 (A5). *n* = 3.

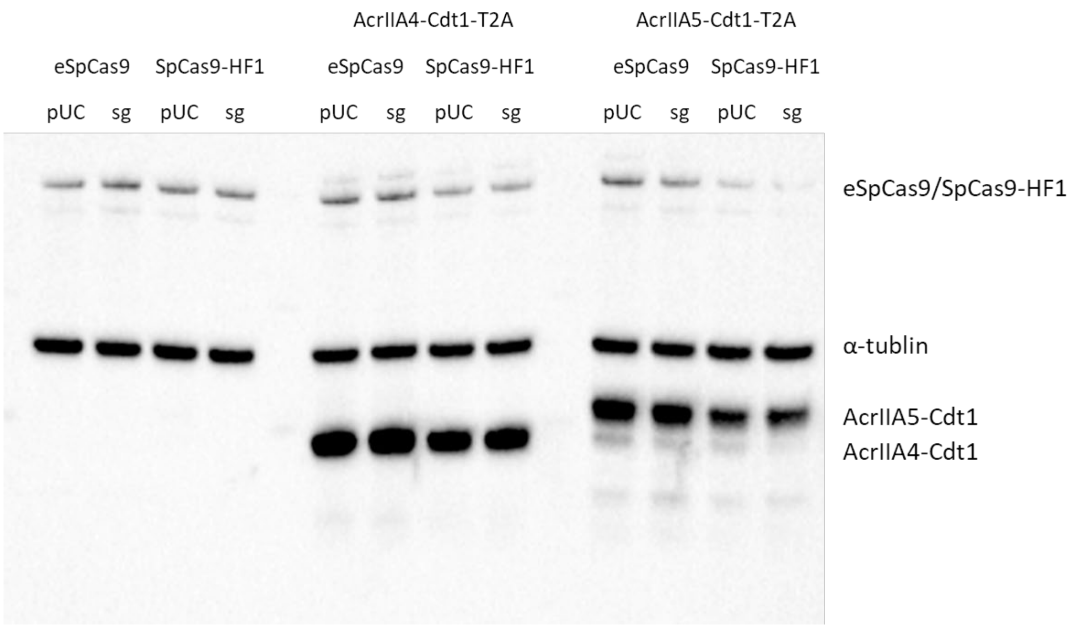

Figure S2. Protein expression levels of eSpCas9, SpCas9-HF1, AcrIIA4-Cdt1, and AcrIIA5-Cdt1 in the presence (sg) or absence (pUC) of sgRNA.

Table S1. All data used in Figure 1. Identical sample names indicate that data were obtained from the same cell population.

HDR	SpCas9	AcrIIA4-Cdt1-T2A-SpCas9	eSpCas9	SpCas9-HF1	HiFi Cas9	HypaCas9	xCas9(3.7)
Sample #1	0.75	3.17	4.20	5.65	0.71	1.19	1.34
Sample #2	1.56	2.91	3.06	4.77	0.64	3.60	1.22
Sample #3	2.25	7.10	7.47	2.70	1.47	4.02	4.46
Sample #4	2.01	5.80	6.19	3.03	2.00	3.32	2.34
Average	1.64	4.75	5.23	4.04	1.21	3.03	2.34
Standard deviation	0.66	2.04	1.98	1.41	0.65	1.26	1.50
p-value compared with Cas9 sample	-	0.03	0.01	0.02	0.38	0.10	0.43

On-target mutation	SpCas9	AcrIIA4-Cdt1-T2A-SpCas9	eSpCas9	SpCas9-HF1	HiFi Cas9	HypaCas9	xCas9(3.7)
Sample #1	80.9	31.5	52	84	73.5	83.1	60.4
Sample #2	59.8	38.6	55.1	87.8	46.7	83.5	73.2
Sample #3	52.4	36.4	69.6	60	68.2	87.8	54.3
Sample #4	90.7	63	70.9	67.1	57.0	79.8	69.9
Average	71.0	42.4	61.9	74.7	61.4	83.6	64.5
Standard deviation	17.9	14.1	9.74	13.3	11.9	3.28	8.67
p-value compared with Cas9 sample	-	0.046	0.408	0.746	0.406	0.215	0.537

Off-target 1 mutation	SpCas9	AcrIIA4-Cdt1-T2A-SpCas9	eCas9	HFCas9	HiFiCas9	HypaCas9	xCas9
Sample #1	79.5	26.9	1.50	0.00	1.60	1.50	5.60
Sample #2	68.4	33.9	0.80	0.00	1.90	0.50	6.60
Sample #3	58	33.6	4.90	2.7	3.60	5.40	7.80
Sample #4	59.9	50.6	5.10	2.90	2.00	3.00	-
Average	66.45	36.3	3.08	1.40	2.28	2.60	6.67
Standard deviation	9.80	10.1	2.24	1.62	0.90	2.13	1.10
p-value compared with Cas9 sample	-	5.14×10^{-3}	1.53×10^{-5}	1.22×10^{-5}	1.26×10^{-5}	1.44×10^{-5}	1.51×10^{-4}

Off-target 2 mutation	SpCas9	AcrIIA4-Cdt1-T2A-SpCas9	eCas9	HFCas9	HiFiCas9	HypaCas9	xCas9
Sample #1	37.1	1.50	1.30	0.00	0.40	0.80	0.50
Sample #2	34.6	9.20	0.50	0.00	1.30	2.00	0.90
Sample #3	25.4	2.40	1.70	2.70	0.90	2.10	0.50
Sample #4	21.5	8.00	0.80	2.90	1.70	1.00	3.60
Average	29.65	5.28	1.08	1.40	1.08	1.48	1.38
Standard deviation	7.40	3.89	0.53	1.62	0.56	0.67	1.50
p-value compared with Cas9 sample	-	1.12×10^{-3}	2.52×10^{-4}	3.00×10^{-4}	2.52×10^{-4}	2.74×10^{-4}	2.94×10^{-4}

HDR	SpCas9	evoCas9	Sniper-Cas9	R63A/Q768A Cas9	LZ3 Cas9	SpartaCas9
Sample #1	1.45	1.29	0.54	0.89	2.23	1.43
Sample #2	0.84	0.88	0.78	0.61	1.88	1.15
Sample #3	1.02	1.33	0.77	0.55	2.97	0.92
Sample #4	1.71	0.83	0.71	0.80	2.55	0.94
Average	1.26	1.08	0.70	0.71	2.41	1.11
Standard deviation	0.396	0.263	0.110	0.158	0.464	0.236
p-value compared with Cas9 sample	-	0.485	0.035	0.0436	0.00933	0.539

On-target mutation	SpCas9	evoCas9	Sniper-Cas9	R63A/Q768A Cas9	LZ3 Cas9	SpartaCas9
Sample #1	79.7	36.1	27.8	91.8	80.9	74.8
Sample #2	79	39.8	24.7	85.8	70.8	78.7
Sample #3	74.2	38.5	29.8	88	71.2	70.7
Sample #4	77.3	38.5	28.7	90	74.4	73.1
Average	77.55	38.23	27.75	88.90	74.33	74.33
Standard deviation	2.450	1.544	2.192	2.585	4.670	3.367
p-value compared with Cas9 sample	-	1.65×10^{-7}	8.58×10^{-8}	7.01×10^{-4}	0.267	0.172

Off-target 1 mutation	SpCas9	evoCas9	Sniper-Cas9	R63A/Q768A Cas9	LZ3 Cas9	SpartaCas9
Sample #1	72.3	7.1	11.4	68.2	0	49.6
Sample #2	77.2	6.4	8.9	72.6	0	42.7
Sample #3	73.9	7.5	10.5	72.6	0	43
Sample #4	72.7	6.5	8.4	74	1.1	4.7
Average	74.03	6.88	9.80	71.85	0.28	35.00
Standard deviation	2.22	0.519	1.39	2.52	0.550	20.45
p-value compared with Cas9 sample	-	1.62×10^{-9}	4.87×10^{-9}	0.243	9.42×10^{-10}	0.00903

Off-target 2 mutation	SpCas9	evoCas9	Sniper-Cas9	R63A/Q768A Cas9	LZ3 Cas9	SpartaCas9
Sample #1	33.7	0	0.2	33.6	4.7	21.8
Sample #2	39.9	0	1.1	31.8	14.9	21.9
Sample #3	32.6	0.4	0.1	30.2	17.1	15.3
Sample #4	33.9	0	0	30.9	5.2	19.8
Average	35.03	0.10	0.35	31.63	10.48	19.70
Standard deviation	3.30	0.200	0.51	1.47	6.446	3.09
p-value compared with Cas9 sample	-	7.33×10^{-7}	8.10×10^{-8}	0.109	5.03×10^{-4}	5.03×10^{-4}

Table S2. All data used in Figure 2. Identical sample names indicate that data were obtained from the same cell population. N.D., not determined.

AAVS1 site

HDR	SpCas9	eSpCas9	AcrIIA4-Cdt1-T2A-eSpCas9	AcrIIA5-Cdt1-T2A-eSpCas9	SpCas9-HF1	AcrIIA4-Cdt1-T2A-SpCas9-HF1	AcrIIA5-Cdt1-T2A-SpCas9-HF1
Sample #1	7.83	7.43	2.63	1.25	5.30	9.87	10.0
Sample #2	5.74	5.29	3.34	1.41	5.56	11.1	7.48
Sample #3	12.4	6.08	8.72	2.68	4.17	6.98	8.73
Average	8.66	6.27	4.90	1.78	5.01	9.32	8.72
Standard deviation	3.41	1.08	3.33	0.79	0.74	2.12	1.24
p-value compared with Cas9 sample	-	0.311	0.243	0.0272	0.144	0.791	0.979

On-target mutation	SpCas9	eCas9	AcrIIA4-Cdt1-T2A-eCas9	AcrIIA5-Cdt1-T2A-eCas9	HF1Cas9	AcrIIA4-Cdt1-T2A-HF1Cas9	AcrIIA5-Cdt1-T2A-HF1Cas9
Sample #1	58.8	57.6	24.4	14.5	40.3	17.9	26.5
Sample #2	61.3	62.6	25.4	13.6	41.2	20.7	27.9
Sample #3	80.9	44.9	24.1	15.7	42.8	17.6	13.5
Average	67.0	55.0	24.6	14.6	41.4	18.7	22.6
Standard deviation	12.1	9.12	0.68	1.05	1.27	1.71	7.94
p-value compared with Cas9 sample	-	0.243	0.00376	0.00172	0.0220	0.00239	0.00605

Off-target mutation	SpCas9	eCas9	AcrIIA4-Cdt1-T2A-eCas9	AcrIIA5-Cdt1-T2A-eCas9	HF1Cas9	AcrIIA4-Cdt1-T2A-HF1Cas9	AcrIIA5-Cdt1-T2A-HF1Cas9
Sample #1	6.70	0.00	0.00	2.80	0.00	0.00	0.00
Sample #2	2.30	0.00	0.00	0.30	0.00	0.00	0.00
Sample #3	4.20	0.50	0.00	0.70	5.00	1.30	0.60
Average	4.40	0.17	0.00	1.27	1.67	0.43	0.20
Standard deviation	2.21	0.29	0.00	1.34	2.89	0.75	0.35
p-value compared with Cas9 sample	-	0.0301	0.0260	0.104	0.263	0.0421	0.0312

HDR	SpCas9	LZ3 Cas9	AcrIIA4-Cdt1-T2A-LZ3 Cas9	AcrIIA5-Cdt1-T2A-LZ3 Cas9
Sample #1	4.80	4.18	1.06	1.30
Sample #2	5.73	3.24	0.05	0.94
Sample #3	5.11	2.66	0.93	0.72
Sample #4	3.07	2.20	0.92	1.21
Average	4.68	3.07	0.74	1.04
Standard deviation	1.142	0.853	0.463	0.263
p-value compared with Cas9 sample	-	0.0650	6.94×10^{-4}	8.14×10^{-4}

On-target mutation	SpCas9	LZ3 Cas9	AcrIIA4-Cdt1-T2A-LZ3 Cas9	AcrIIA5-Cdt1-T2A-LZ3 Cas9
Sample #1	55.3	37.2	5.1	4.5

Sample #2	48.3	41	0	3.1
Sample #3	56.5	40	4.5	3.1
Sample #4	27.9	30.7	3.3	3.2
Average	47.0	37.2	3.23	3.48
Standard deviation	13.2	4.64	2.28	0.685
p-value compared with Cas9 sample	-	0.213	6.22×10^{-4}	5.97×10^{-4}

Off-target mutation	SpCas9	LZ3 Cas9	AcrIIA4-Cdt1-T2A-LZ3 Cas9	AcrIIA5-Cdt1-T2A-LZ3 Cas9
Sample #1	5.50	2.00	0.70	0.50
Sample #2	2.80	0.70	0.30	0.60
Sample #3	0.40	0.80	0.80	0.60
Sample #4	0.90	2.30	0.50	0.60
Average	4.40	0.17	0.00	1.27
Standard deviation	2.21	0.29	0.00	1.34
p-value compared with Cas9 sample	-	2.31	0.819	0.222

EMX1 site

HDR	SpCas9	eSpCas9	AcrIIA4-Cdt1-T2A-eSpCas9	AcrIIA5-Cdt1-T2A-eSpCas9	SpCas9-HF1	AcrIIA4-Cdt1-T2A-SpCas9-HF1	AcrIIA5-Cdt1-T2A-SpCas9-HF1
Sample #1	0.75	6.04	3.58	0.68	5.65	5.79	0.57
Sample #2	1.56	9.12	4.03	0.63	4.77	6.47	0.40
Sample #3	1.07	2.50	1.82	0.00	2.84	5.75	0.00
Sample #4	0.98	4.96	2.08	0.00	2.22	6.05	0.00
Average	1.09	5.65	2.88	0.33	3.87	6.01	0.24
Standard deviation	0.34	2.75	1.09	0.38	1.61	0.33	0.29
p-value compared with Cas9 sample	-	0.0164	0.0203	0.0247	0.0148	8.17×10^{-7}	0.00896

On-target mutation	SpCas9	eSpCas9	AcrIIA4-Cdt1-T2A-eSpCas9	AcrIIA5-Cdt1-T2A-eSpCas9	SpCas9-HF1	AcrIIA4-Cdt1-T2A-SpCas9-HF1	AcrIIA5-Cdt1-T2A-SpCas9-HF1
Sample #1	78.9	78.5	34.8	8.2	84.0	62.3	27.8
Sample #2	83.2	88.1	29.0	11.3	87.8	64.7	28.0
Sample #3	80.9	80.9	26.1	11.0	60.0	38.6	25.6
Sample #4	59.8	79.9	16.0	8.8	67.1	22.0	29.4
Average	75.70	81.85	26.48	9.83	74.73	46.90	27.70
Standard deviation	10.74	4.28	7.86	1.55	13.32	20.35	1.57
p-value compared with Cas9 sample	-	0.328	3.14×10^{-4}	1.90×10^{-5}	0.9130	0.0464	1.16×10^{-4}

Off-target 1 mutation	SpCas9	eCas9	AcrIIA4-Cdt1-T2A-eCas9	AcrIIA5-Cdt1-T2A-eCas9	HF1Cas9	AcrIIA4-Cdt1-T2A-HF1Cas9	AcrIIA5-Cdt1-T2A-HF1Cas9
Sample #1	82.5	1.00	0.00	0.20	0.00	0.20	0.00
Sample #2	92.5	0.00	0.10	0.00	0.00	2.30	0.00
Sample #3	79.5	1.50	1.80	1.60	2.70	2.00	2.70
Sample #4	68.4	0.60	0.70	1.90	2.90	1.50	1.80
Average	80.73	0.78	0.65	0.93	1.40	1.50	1.13
Standard deviation	9.92	0.63	0.83	0.96	1.62	0.93	1.35
p-value compared with Cas9 sample	-	3.67×10^{-6}	3.66×10^{-6}	3.77×10^{-6}	4.10×10^{-6}	3.923×10^{-6}	3.92×10^{-6}

Off-target 2 mutation	SpCas9	eCas9	AcrIIA4-Cdt1-T2A-eCas9	AcrIIA5-Cdt1-T2A-eCas9	HF1Cas9	AcrIIA4-Cdt1-T2A-HF1Cas9	AcrIIA5-Cdt1-T2A-HF1Cas9
Sample #1	55.5	0.00	1.40	0.00	0.00	0.00	0.00
Sample #2	53.7	0.00	2.10	1.90	0.00	0.00	0.50
Sample #3	37.1	0.00	0.10	0.60	2.70	0.60	1.50
Sample #4	34.6	0.20	0.00	2.20	2.90	0.00	1.40
Average	45.23	0.05	0.90	1.18	1.40	0.15	0.85
Standard deviation	10.90	0.10	1.02	1.05	1.62	0.30	0.72
p-value compared with Cas9 sample	-	1.67×10^{-4}	1.90×10^{-4}	1.97×10^{-4}	2.10×10^{-4}	1.69×10^{-4}	1.87×10^{-4}

HDR	SpCas9	LZ3 Cas9	AcrIIA4-Cdt1-T2A-LZ3 Cas9	AcrIIA5-Cdt1-T2A-LZ3 Cas9
Sample #1	1.449	1.854	1.446	0.284
Sample #2	0.844	3.293	1.339	0.774
Sample #3	1.023	1.613	1.313	0.829
Sample #4	1.713	2.701	1.086	0.523
Average	1.26	2.37	1.30	0.60
Standard deviation	0.396	0.775	0.151	0.251
p-value compared with Cas9 sample	-	0.0437	0.861	0.0314

On-target mutation	SpCas9	LZ3 Cas9	AcrIIA4-Cdt1-T2A-LZ3 Cas9	AcrIIA5-Cdt1-T2A-LZ3 Cas9
Sample #1	72.3	56.4	11.5	0.40
Sample #2	79	58.1	12.5	5.00
Sample #3	74.2	46.6	10.3	4.60
Sample #4	77.3	69.6	9.90	3.50
Average	75.70	57.7	11.1	3.38
Standard deviation	3.014	9.43	1.18	2.08
p-value compared with Cas9 sample	-	0.0108	1.65×10^{-8}	1.76×10^{-8}

Off-target 1 mutation	SpCas9	LZ3 Cas9	AcrIIA4-Cdt1-T2A-LZ3 Cas9	AcrIIA5-Cdt1-T2A-LZ3 Cas9
Sample #1	72.3	17.2	6.20	6.50
Sample #2	77.2	7.60	6.80	7.90
Sample #3	73.9	12.4	6.80	6.50
Sample #4	72.7	8.50	6.20	6.20

Average	74.0	11.4	6.50	6.78
Standard deviation	2.22	4.38	0.35	0.76
p-value compared with Cas9 sample	-	2.40×10^{-7}	1.44×10^{-9}	1.91×10^{-9}

Off-target 2 mutation	SpCas9	LZ3 Cas9	AcrIIA4-Cdt1-T2A-LZ3 Cas9	AcrIIA5-Cdt1-T2A-LZ3 Cas9
Sample #1	33.7	5.50	0	0
Sample #2	39.9	0	2.70	0.70
Sample #3	32.6	0	0	0
Sample #4	33.9	0.10	0.10	0
Average	35.03	1.40	0.70	0.18
Standard deviation	3.30	2.73	1.33	0.350
p-value compared with Cas9 sample	-	4.24×10^{-6}	1.26×10^{-6}	7.59×10^{-7}

VEGFA site

HDR	SpCas9	eSpCas9	AcrIIA4-Cdt1-T2A-eSpCas9	AcrIIA5-Cdt1-T2A-eSpCas9	SpCas9-HF1	AcrIIA4-Cdt1-T2A-SpCas9-HF1	AcrIIA5-Cdt1-T2A-SpCas9-HF1
Sample #1	1.68	2.43	3.25	0.00	1.54	3.65	2.63
Sample #2	2.56	2.57	3.43	0.00	1.75	4.33	2.30
Sample #3	2.79	2.90	3.64	0.00	1.82	3.50	2.24
Average	2.35	2.63	3.44	0.00	1.70	3.83	2.39
Standard deviation	0.59	0.24	0.19	0.00	0.15	0.44	0.21
p-value compared with Cas9 sample	-	0.476	0.0377	0.00233	0.142	0.0250	0.909

On-target mutation	SpCas9	eSpCas9	AcrIIA4-Cdt1-T2A-eSpCas9	AcrIIA5-Cdt1-T2A-eSpCas9	SpCas9-HF1	AcrIIA4-Cdt1-T2A-SpCas9-HF1	AcrIIA5-Cdt1-T2A-SpCas9-HF1
Sample #1	72.7	80.3	23.1	0.90	69.8	34.8	24.6
Sample #2	71.2	74.8	26.8	2.5	64.3	30.8	25.4
Sample #3	68.2	71.4	20.6	0.70	64.0	34.4	24.1
Average	70.70	75.50	23.50	1.37	66.03	33.33	24.70
Standard deviation	2.29	4.49	3.12	0.99	3.27	2.20	0.66
p-value compared with Cas9 sample	-	0.174	2.97×10^{-5}	1.11×10^{-6}	0.113	3.44×10^{-5}	4.78×10^{-6}

Off-target 1 mutation	SpCas9	eSpCas9	AcrIIA4-Cdt1-T2A-eSpCas9	AcrIIA5-Cdt1-T2A-eSpCas9	SpCas9-HF1	AcrIIA4-Cdt1-T2A-SpCas9-HF1	AcrIIA5-Cdt1-T2A-SpCas9-HF1
Sample #1	59.2	52.7	9.90	0.50	12.0	17.9	2.70
Sample #2	64.6	48.8	13.6	1.90	15.0	13.5	2.00
Sample #3	64.3	49.0	12.0	10.5	11.8	3.50	7.60
Average	62.7	50.2	11.8	4.30	12.9	11.6	4.10
Standard deviation	3.03	2.20	1.86	5.41	1.79	7.38	3.05
p-value compared with Cas9 sample	-	0.00440	1.58×10^{-5}	8.30×10^{-5}	1.65×10^{-5}	3.77×10^{-4}	1.92×10^{-5}

Off-target 2 mutation	SpCas9	eSpCas9	AcrIIA4-Cdt1-T2A-eSpCas9	AcrIIA5-Cdt1-T2A-eSpCas9	SpCas9-HF1	AcrIIA4-Cdt1-T2A-SpCas9-HF1	AcrIIA5-Cdt1-T2A-SpCas9-HF1
Sample #1	32.9	0.00	0.70	5.70	6.70	9.90	7.00
Sample #2	38.9	7.30	5.60	5.20	6.60	7.60	5.50
Sample #3	35.3	6.40	5.30	0.70	5.20	5.00	5.40
Average	35.7	4.57	3.87	3.87	6.17	7.50	5.97
Standard deviation	3.02	3.98	2.75	2.75	0.84	2.45	0.90
p-value compared with Cas9 sample	-	4.18×10^{-4}	1.74×10^{-4}	1.75×10^{-4}	8.25×10^{-5}	2.31×10^{-4}	8.19×10^{-5}

HDR	SpCas9	LZ3 Cas9	AcrIIA4-Cdt1-T2A-LZ3 Cas9	AcrIIA5-Cdt1-T2A-LZ3 Cas9
Sample #1	3.99	5.85	2.86	2.28
Sample #2	4.43	5.35	2.82	1.83
Sample #3	4.25	5.11	4.01	1.83
Average	4.22	5.44	3.23	1.98
Standard deviation	0.223	0.376	0.676	0.257
p-value compared with Cas9 sample	-	0.00857	0.0739	3.36×10^{-4}

On-target mutation	SpCas9	LZ3 Cas9	AcrIIA4-Cdt1-T2A-LZ3 Cas9	AcrIIA5-Cdt1-T2A-LZ3 Cas9
Sample #1	65.2	56.5	9.5	6.2
Sample #2	62.4	53.7	7.8	6.4
Sample #4	56.1	47.5	5.7	4.0
Average	61.23	52.57	7.67	5.53
Standard deviation	4.66	4.61	1.90	1.33
p-value compared with Cas9 sample	-	0.0838	5.10×10^{-5}	3.76×10^{-5}

Off-target 1 mutation	SpCas9	LZ3 Cas9	AcrIIA4-Cdt1-T2A-LZ3 Cas9	AcrIIA5-Cdt1-T2A-LZ3 Cas9
Sample #1	53.3	31.4	2.8	0.6
Sample #2	51.4	28.2	1.7	9.6
Sample #3	55.2	26.4	3.0	2.4
Average	53.3	28.7	2.5	4.2
Standard deviation	1.90	2.53	0.70	4.76
p-value compared with Cas9 sample	-	1.75×10^{-4}	1.68×10^{-6}	7.74×10^{-5}

Off-target 2 mutation	SpCas9	LZ3 Cas9	AcrIIA4-Cdt1-T2A-LZ3 Cas9	AcrIIA5-Cdt1-T2A-LZ3 Cas9
Sample #1	23	0.10	0	0.50
Sample #2	29.5	0.40	0	0.10
Sample #4	24.8	1.00	0	0.00
Average	25.8	0.50	0	0.20
Standard deviation	3.36	0.46	0	0.265
p-value compared with Cas9 sample	-	2.07×10^{-4}	1.84×10^{-4}	1.93×10^{-4}

Table S3. Sequences for ssODNs and primers

Primer Name	Sequence (5' to 3')	Description
**For vector construction**		
NLS-Acr-Cdt1 long Fw	ccgggggatccactagttctagagcGCCACCATGGACAAGAAGTAC	for construction of pEB.AcrIIA4/AcrIIA5-Cdt1-T2A-Cas9 variants (HiFiCas9, xCas9, evoCas9, SniperCas9, LZ3 Cas9, R63A/Q768A Cas9, and SpartaCas9)
pEBCas9 Fw	ccgggggatccactagttctagagcGCCACCATGGACAAGAAGTAC	for construction of pEB.HiFiCas9 and pEB.xCas9
pEBCas9 Rv	atggctgattatgatctagagtcgcTCACACCTTCCTCTTCTTCTTGG	for construction of pEB.HiFiCas9, xCas9, evoCas9, SniperCas9, LZ3 Cas9, R63A/Q768A Cas9, and SpartaCas9, pEB.AcrIIA4/AcrIIA5-Cdt1-T2A- (HiFiCas9, xCas9, evoCas9, SniperCas9, LZ3 Cas9, R63A/Q768A Cas9, and SpartaCas9)
HiFi mut R691A Fw	AAGTCCGATGGATTTGCCAACgccAACTTCATGCAGTTGATCCA	for construction pEB.HiFiCas9
HiFi mut R691A Rv	TGGATCAACTGCATGAAGTTggcGTTGGCAAATCCATCGGACTT	for HiFi Cas9 mutant plasmid
NLS-AcrCdt1-HypaHF long Fw	ccaggacaccatcgagggcagaggaagtctgctaacatgcggtgacgtcgaggagaatcctggccca gataaaaagtattctattggttta	for construction of pEB.AcrIIA4-Cdt1-T2A-HF1Cas9, pEB.AcrIIA5-Cdt1-T2A-HF1Cas9, pEB.AcrIIA4-Cdt1-T2A-HypaCas9, and pEB.AcrIIA5-Cdt1-T2A-HypaCas9
pEBHypaHFCas9 Fw	ccgggggatccactagttctagagc GCCACCATG gataaaaagtattctattggttta	for construction of pEB.HF1Cas9 and pEB.HypaCas9
pEBHypaHFCas9 Rv	atggctgattatgatctagagtcgcTCAgactttcctcttcttcttggg	for construction of pEB.HF1Cas9, pEB.AcrIIA4-T2A-HF1Cas9, pEB.AcrIIA5-T2A-HF1Cas9, pEB.AcrIIA4-Cdt1-T2A-HF1Cas9, pEB.AcrIIA5-Cdt1-T2A-HF1Cas9, pEB.HypaCas9, pEB.AcrIIA4-T2A-HypaCas9, pEB.AcrIIA5--T2A-HypaCas9, pEB.AcrIIA4-Cdt1-T2A-HypaCas9, and pEB.AcrIIA5-Cdt1-T2A-HypaCas9
NLS-AcrCdt1-eSp long Fw	ccaggacaccatcgagggcagaggaagtctgctaacatgcggtgacgtcgaggagaatcctggccca gacaagaagtacagcatcgg	for construction of pEB.AcrIIA4-Cdt1-T2A-eCas9 and pEB.AcrIIA5-Cdt1-T2A-eCas9
pEBeSpCas9 Fw	ccgggggatccactagttctagagc GCCACCATG gacaagaagtacagcatcgg	for construction of pEB.eCas9
pEBeSpCas9 Rv	atggctgattatgatctagagtcgcTCA gactttcctcttcttcttggggtcgcctcccagctgagac	for construction of pEB.eCas9, pEB.AcrIIA4-T2A-eCas9, pEB.AcrIIA5-T2A-eCas9, pEB.AcrIIA4-Cdt1-T2A-eCas9, and pEB.AcrIIA5-Cdt1-T2A-eCas9
GA pEB NLS Fw	GGATCCACTAGTTCTAGAGCGCCACCATGGGTGGCCCG	for construction of AcrIIA4, AcrIIA5, AcrIIA4-Cdt1,and AcrIIA5-Cdt1 fragments
T2A Rv	TGGGCCAGGATTCTCCTCGA	for construction of AcrIIA4-Cdt1and AcrIIA5-Cdt1 fragments
FLAG Rv for Cdt1 remove	GACTTCCTCTGCCCTCCTTATCGTCATCGTCCTTGTAGTC	for construction of AcrIIA4 and AcrIIA5 fragments
5 end T2A Fw	GAGGGCAGAGGAAGTCTGCT	for construction of pEB-AcrIIA4/5-T2A-Cas9mutants
M495V/Y515N/K526E Fw for evoCas9	CATCGAAAGGgtgACTAACTTTGATAAAAATCTGCCTAACGAAAAGGTGCTTCCTAAACACTCTCTGCTG	for construction of pEB.evoCas9
M495V Rv for evoCas9	CAAAGTTAGTCAcCCTTTCGATGAAGGACTGGG	for construction of pEB.evoCas9
M495V/Y515N/K526E Rv for evoCas9	TCTGTGACGTATTTGACctcGGTGAGCTCGTTATAAACTGTGAAGTACTCgttCAGCAGAGAGTGTTTAGGAA	for construction of pEB.evoCas9
K526E Fw for evoCas9	TAACGAGCTCACCgagGTCAAATACGTCACAGA	for construction of pEB.evoCas9
R661L Rv for evoCas9	TTCTTGACAGaagCCCCCATCCTGTATATCGGC	for construction of pEB.evoCas9
R661L Fw for evoCas9	AGGATGGGGGcttCTGTCAAGAAAACTGATCAA	for construction of pEB.evoCas9
F539S Rv for Sniper-Cas9	CTCCAGACAGggaTGCTGGCTTTCTCATCCCTT	for construction of pEB.Sniper-Cas9
F539S Fw for Sniper-Cas9	AAAGCCAGCAtccCTGTCTGGAGAGCAGAAGAA	for construction of pEB.Sniper-Cas9
M763I Rv for Sniper-Cas9	TCTCTCGGGCtatCTCGATAACGATATTCTCGG	for construction of pEB.Sniper-Cas9
M763I Fw for Sniper-Cas9	CGTTATCGAGataGCCCGAGAGAACCAAACTAC	for construction of pEB.Sniper-Cas9
K890N Rv for Sniper-Cas9	GTGTGATCAGattGGCGTTCAGCAGCTGCCGCC	for construction of pEB.Sniper-Cas9
K890N Fw for Sniper-Cas9	GCTGAACGCCaatCTGATCACACAACGGAAGTT	for construction of pEB.Sniper-Cas9
N690C Rv for LZ3 Cas9	GAAGTTCCGgcaGGCAAATCCATCGGACTTAAG	for construction of pEB.LZ3 Cas9
N690C Fw for LZ3 Cas9	TGGATTTGCCtgcCGGAACTTCATGCAGTTGAT	for construction of pEB.LZ3 Cas9
T769I Rv for LZ3 Cas9	CCTTCTGGGTtatTTGGTTCTCTCGGGCCATCT	for construction of pEB.LZ3 Cas9
T769I Fw for LZ3 Cas9	AGAGAACCAAataACCCAGAAGGGACAGAAGAA	for construction of pEB.LZ3 Cas9
G915M Rv for LZ3 Cas9	TTTTGATGAAcatGGCTTTATCCAACTCAGACA	for construction of pEB.LZ3 Cas9
G915M Fw for LZ3 Cas9	GGATAAAGCCatgTTCATCAAAAGGCAGCTTGT	for construction of pEB.LZ3 Cas9
N980K Rv for LZ3 Cas9	CATGGTGGTActtGTTGATCTCTCTCACCTTAT	for construction of pEB.LZ3 Cas9
N980K Fw for LZ3 Cas9	AGAGATCAACaagTACCACCATGCGCATGATGC	for construction of pEB.LZ3 Cas9
R63A Rv for R63A/Q768A Cas9	TTCTTTTGAGcgcCGTGGCTTCGGCCGTCTCCC	for construction of pEB.R63A/Q768A Cas9
R63A Fw for R63A/Q768A Cas9	CGAAGCCACGgcgCTCAAAAGAACAGCACGGCG	for construction of pEB.R63A/Q768A Cas9
Q768A Rv for R63A/Q768A	TCTGGGTAGTtgcGTTCTCTCGGGCCATCTCGA	for construction of pEB.R63A/Q768A Cas9
Q768A Fw for R63A/Q768A	CCGAGAGAACgcaACTACCCAGAAGGGACAGAA	for construction of pEB.R63A/Q768A Cas9
D23A Rv for SpartaCas9	CCTTGTACTCtgcCGTAATGACGGCCCAGCCGA	for construction of pEB.SpartaCas9
D23A Fw for SpartaCas9	CGTCATTACGgcaGAGTACAAGGTGCCGAGCAA	for construction of pEB.SpartaCas9
T67L Rv for SpartaCas9	TGCGCCGTGCcagTCTTTTGAGCCGCGTGGCTT	for construction of pEB.SpartaCas9
T67L Fw for SpartaCas9	GCTCAAAAGActgGCACGGCGCAGATATACCCG	for construction of pEB.SpartaCas9
Y128V Rv for SpartaCas9	ACTTTTCATGgacCGCCACCTCGTCCACGATAT	for construction of pEB.SpartaCas9
Y128V Fw for SpartaCas9	CGAGGTGGCGgtcCATGAAAAGTACCCAACCAT	for construction of pEB.SpartaCas9
D1251G Rv for SpartaCas9	TCTGCTCATTaccTTCGGGAGACCCTTTGAGCT	for construction of pEB.SpartaCas9
D1251G Fw for SpartaCas9	GTCTCCCGAAggtAATGAGCAGAAGCAGCTGTT	for construction of pEB.SpartaCas9
**For editing assesment**		
AAVS1 ssODN	TATATTCCCAGGGCCGGTTAATGTGGCTCTGGTTCTGGGTACTTTTATCTGTCCCCTCCGGATCCAAGCTTCCACAGTGGGGCCACTAGGGACAGGATTGGTGACAGAAAAGCCCCATCCTTAGGCCTCC	for HDR template
AAVS1 target Fw	CCTCTCTGGCTCCATCGTAAGCAAA	for AAVS1 target site amplification
AAVS1 target Rv	CCGAGAGCTCAGCTAGTCTTCTTCC	for AAVS1 target site amplification
AAVS1 off-target Fw	GGGTGGGGATGCATTAGGGCTTGGT	for AAVS1 off-target site amplification
AAVS1 off-target Rv	CCCCACCCAGACATAGAACCCTTTGC	for AAVS1 off-target site amplification
EMX1 ssODN	TCCTCCCCATTGGCCTGCTTCGTGGCAATGCGCCACCGGTTGATGTGATGGGAGCCCTTGGATCCAAGCTTCTTCTGCTCGGACTCAGGCCCTTCCTCCTCCAGCTTCTGCCGTTTGTACTTTGTCCTC	for HDR template
EMX1 target Fw	GCCCTGCCATCCCCTTCTGTGAATG	for EMX1 target site amplification
EMX1 target Rv	CCAGGCCTCAGCCAGCCCATTGCTT	for EMX1 target site amplification
EMX1 off-target 1 Fw	GCCCCTCTAATACAATGGGAAGGACAGCTT	for EMX1 Off-target site 1 amplification
EMX1 off-target 1 Rv	GGGCCTGCAGACAGGAATAGCCCTA	for EMX1 Off-target site 1 amplification
EMX1 off-target 2 Fw	GGCTGCCATGGAAATTCCAGAGGGA	for EMX1 Off-target site 2 amplification
EMX1 off-target 2 Rv	GCCGGGGGTCACTGGATGAATCCTC	for EMX1 Off-target site 2 amplification
VEGFA ssODN	TATTGGAATCCTGGAGTGACCCCTGGCCTTCTCCCCGCTCCAACGCCCTCAACCCCACAGGATCCAAGCTTACACACTCACTCACCCACACAGACACACACGTCCTCACTCTCGAAGACGCTGCTCGCT	for HDR template

VEGFA target Fw	GCCGCTCACTTTGATGTCTG	for VEGFA target site amplification
VEGFA target Rv	GAGCCGTTCCCTCTTTGCTA	for VEGFA target site amplification
VEGFA off-target 1 Fw	AACTACAAGTCTCGGCTGCC	for VEGFA Off-target site 1 amplification
VEGFA off-target 1 Rv	CCCAACCTCCAGTCCCAGG	for VEGFA Off-target site 1 amplification
VEGFA off-target 2 Fw	AGCGATTCTCCTGCCTCAACC	for VEGFA Off-target site 2 amplification
VEGFA off-target 2 Rv	CAGTATGAACGGGAGTGGTAAGGA	for VEGFA Off-target site 2 amplification
